# A zebrafish luminescent biosensor for kidney tubulopathy, metal toxicity and drug screening

**DOI:** 10.1242/dmm.052673

**Published:** 2026-05-22

**Authors:** Han Lai, Monica Goldade, Svenja Aline Keller, Isabelle Worms, Alessandro Luciani, Stephan C. F. Neuhauss, Vera I. Slaveykova, Olivier Devuyst, Zhiyong Chen

**Affiliations:** ^1^ Department of Physiology, University of Zurich, 8057 Zurich, Switzerland; ^2^ Department of Nephrology, First Affiliated Hospital of Chongqing Medical University, Chongqing 400016, China; ^3^ Department F.-A. Forel for Environmental and Aquatic Sciences, School of Earth and Environmental Sciences, Faculty of Science, University of Geneva, CH-1211 Geneva 4, Switzerland; ^4^ Department of Molecular Life Sciences, University of Zurich, 8057 Zurich, Switzerland

**Keywords:** Proximal tubule, Low-molecular-weight proteinuria, NanoLuc luciferase, Receptor-mediated endocytosis, Lysosome

## Abstract

An efficient endolysosomal pathway is crucial to mediate the reabsorption and processing of ultrafiltered solutes including low-molecular-weight (LMW) proteins by epithelial cells lining the proximal tubule (PT) of the kidney. The zebrafish pronephros is used as a model system for congenital or acquired disorders that impair endolysosomal processing in PT cells, causing inappropriate loss of solutes and LMW proteins in urine. Here, we describe a new reporter *½vdbp-NanoLuc* zebrafish line, in which vitamin D-binding protein is coupled to NanoLuc luciferase for detection of PT dysfunction and LMW proteinuria. We demonstrate the reliability and value of the *½vdbp-NanoLuc* biosensor in fish models of monogenic endolysosomal diseases, gentamicin and cisplatin-induced nephrotoxicity, and metal contamination. This novel reporter system yields mechanistic insights into cadmium- and copper-induced PT dysfunction and provides a platform for drug screening.

## Teaser

**Summary:** A zebrafish luminescent biosensor has been generated, suitable to model genetic kidney diseases, to detect nephrotoxicity and metal contamination, and to use for drug screening for endolysosomal diseases.

## INTRODUCTION

The epithelial cells lining the proximal tubule (PT) of the kidney play a key role in controlling body homeostasis through their capacity to reabsorb a large variety of solutes. The latter include low-molecular-weight (LMW) proteins such as hormones, vitamins and their binding proteins, enzymes, immunoglobulin light chains, drugs and toxins, which are ultrafiltered but not, or minimally, excreted in the final urine. Receptor-mediated endocytosis and the associated lysosome–autophagy pathway are essentials for the reabsorptive and processing functions of PT cells and for maintaining homeostasis (van der Wijst et al., 2019). Congenital or acquired disorders, as well as exposure to drugs and toxins, may impair the endolysosomal machinery in PT cells, causing defective reabsorption and inappropriate loss of LMW proteins and vital nutrients into the urine (Devuyst and Luciani, 2015; Festa et al., 2023; van der Wijst et al., 2019). The kidney PT is also the main site of accumulation and toxicity of metals such as cadmium (Cd), copper (Cu), mercury and lead (Pb), which are extensively used in agriculture and industrial activities (Lentini et al., 2017; Rawee et al., 2023). Clinical and experimental studies have demonstrated that detection of LMW proteinuria is the most consistent and sensitive approach to detect congenital and acquired PT dysfunction (Bernard and Lauwerys, 1995; Bökenkamp, 2020; Devuyst, 2018; Hall et al., 2022).

The zebrafish has emerged as a prominent model for studying human kidney disease and chemical nephrotoxicity owing to its highly conserved nephron segment patterning (Outtandy et al., 2018; Poureetezadi and Wingert, 2016). In particular, PT cells of the zebrafish pronephros display a high endocytic uptake mediated by the endocytic receptors megalin and cubilin – similar to that observed in the human kidney. The generation of zebrafish models for Donnai–Barrow syndrome (Veth et al., 2011), Lowe syndrome (Oltrabella et al., 2015) and cystinosis (Festa et al., 2018) indicated the value of the zebrafish system to investigate mechanisms and potential therapeutic targets for endolysosomal disorders of the kidney.

Standard assessment of PT function in zebrafish has long relied on visualizing uptake of injected LMW fluorescent tracers. These assays are labor intensive, challenging to quantify and limited by poor sensitivity, elevated background levels, and difficulty to perform accurate and reproducible intravenous tracer injection. These limitations restrict their use for large-scale assessment of PT dysfunction, e.g. for drug discovery and toxicology screens. To overcome this barrier, we previously developed a transgenic *½vdbp-mCherry* reporter zebrafish line for monitoring LMW proteinuria based on quantifying the vitamin D-binding protein (VDBP)-mCherry in urine by enzyme-linked immunosorbent assay (ELISA) (Chen et al., 2020). Although this line was validated to assess PT dysfunction in endolysosomal disorders (Chen et al., 2020), its scope and applicability is limited owing to the narrow dynamic range, cost and time-intensive nature of the assay.

Luminometry is increasingly being used to overcome the limitations of ELISAs, owing to its higher sensitivity, broad dynamic range and simpler handing (Hall et al., 2021; Jackson et al., 1996). Here, we hypothesized that a NanoLuc-based LMW proteinuria reporter will be superior to an ELISA-reliant system for detecting PT dysfunction in various settings. We established a new *½vdbp-NanoLuc* zebrafish biosensor for LMW proteinuria, by coupling VDBP to NanoLuc luciferase (England et al., 2016), the catalytic subunit of luciferase isolated from *Oplophorus gracilirostris* (Inouye et al., 2000). We validated the biosensor using genetic models of endolysosomal diseases (Chen et al., 2020; Festa et al., 2018), and nephrotoxins including metal pollutants (Hentschel et al., 2005; Kim et al., 2020; Rawee et al., 2023), and demonstrated its value for drug screening. These studies establish the *½vdbp-NanoLuc* biosensor fish system as a platform to investigate mechanisms and potential treatments of congenital and acquired proximal tubulopathies of the kidney.

## RESULTS

### Establishment of the *½vdbp-NanoLuc* zebrafish reporter system

**Fig. 1.**
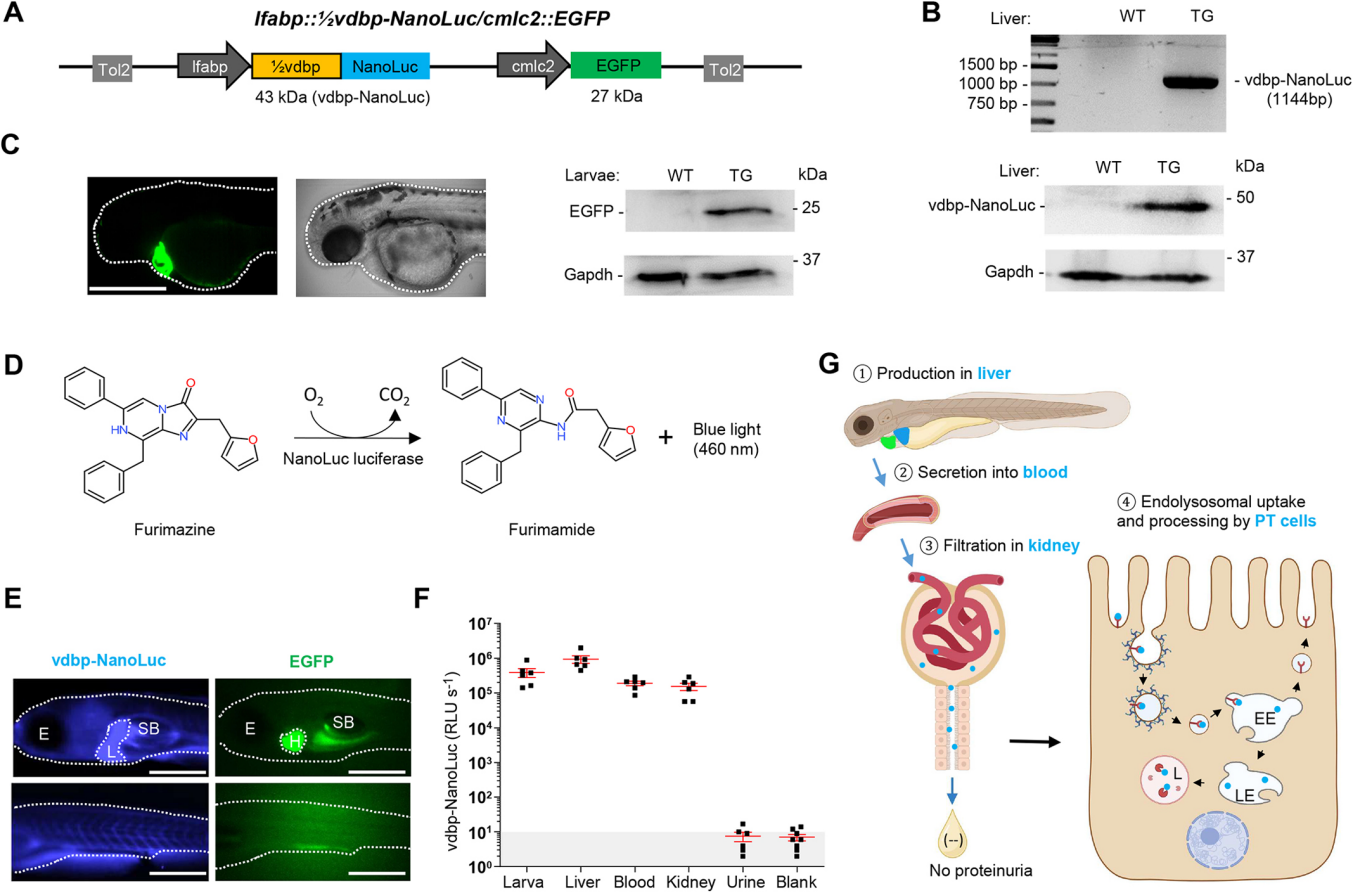
**Establishment of the *½vdbp-NanoLuc* zebrafish reporter system.** (A) Construction of plasmid vector *lfabp::½vdbp-NanoLuc* containing the transgenesis marker *cmlc2*:*:EGFP* and Tol2 transposon elements. The N-terminal 207 amino acids of zebrafish VDBP (½vdbp) are fused with NanoLuc luciferase, which is predicted to have a molecular weight of 43 kDa. The liver fatty acid binding protein (*lfabp*) promoter drives the expression of vdbp-NanoLuc in the liver, and the cardiac myosin light chain 2 (*cmlc2*; also known as *myl7*) promoter controls the expression of EGFP in the heart. Tol2, transposable elements facilitating the integration of plasmid DNA into the embryonic genome. (B) Analysis of *½vdbp-NanoLuc* cDNA (1144 bp) after amplification by polymerase chain reaction (top) and immunoblotting analysis of vdbp-NanoLuc (bottom) in liver lysate. *n*=3 replicates. TG, transgenic zebrafish; WT, wild type. (C) Observation of EGFP fluorescence in the heart of transgenic larvae visualized at 2 days post-fertilization (dpf) by light-sheet microcopy (left) and detection of EGFP (27 kDa) by immunoblotting in larval lysate from WT and transgenic *½vdbp-NanoLuc* larvae (right). *n*=3 replicates; one sample is a pool of five larvae. Dotted lines outline the larva head/body. Scale bar: 0.5 mm. (D) The oxidation of furimazine by NanoLuc luciferase in the presence of O_2_ produces furimamide, CO_2_ and blue light with a wavelength of 460 nm. (E) Observation of *½vdbp-NanoLuc* larva at 5 dpf by bioluminescence microscopy using an Olympus LV200. With incubation of the furimazine substrate, bioluminescent light from vdbp-NanoLuc (blue) is seen at least in the liver and vessels, with EGFP fluorescence (green) visible in the heart. *n*=3. Dotted lines outline the larva head/body, liver and heart. E, eye; H, heart; L, liver; SB, swim bladder. Scale bars: 0.5 mm. (F) Quantification of vdbp-NanoLuc luciferase activity in transgenic zebrafish by luminometry: whole larval lysates (lane 1), isolated liver (lane 2), blood (lane 3), isolated kidney (lane 4), urine collected from 5 dpf larvae (lane 5), blank (E3 medium, lane 6); *n*=6. (G) Schematic illustration of the fate of recombinant protein vdbp-NanoLuc under normal physiological conditions: vdbp-NanoLuc is produced in liver ①, secreted into the bloodstream ②, passed through the glomerulus filtration in kidney ③, reabsorbed and processed by proximal tubule epithelial cells ④. EE, early endosome; LE, late endosome; L, lysosome; PT, proximal tubule.

The novel bioluminescent reporter line is based on VDBP, a physiological LMW ligand for megalin, coupled to NanoLuc luciferase. The expression vector includes the 24 kDa, 207 N-terminal amino acids of zebrafish VDBP tagged with 19 kDa NanoLuc luciferase (termed vdbp-NanoLuc) for expression in liver under control of the *fabp10a* (also known as *lfabp*) promoter and the transgenesis marker *cmlc2::EGFP* for expression of EGFP in the heart (Fig. 1A), allowing sorting of embryos by EGFP fluorescence (Huang et al., 2003). The resulting transgenic *Tg(lfabp::½vdbp-NanoLuc/cmlc2::EGFP)* zebrafish larvae (termed *½vdbp-NanoLuc* line) expressed the 43 kDa vdbp-NanoLuc protein in liver (Fig. 1B) and EGFP in heart (Fig. 1C).

NanoLuc luciferase catalyzes the oxidation of furimazine and related substrates including hikarazine Z108, yielding a blue light at 460 nm (Fig. 1D; Fig. S1A), with an intensity directly proportional to the concentration of NanoLuc luciferase (Fig. S1B,C). Under a bioluminescence microscope, blue light in liver and blood vessels of 5 days post-fertilization (dpf) *½vdbp-NanoLuc* larvae was observed in the presence of furimazine, with EGFP expression in the heart (Fig. 1E). The vdbp-NanoLuc protein was detected in liver, blood and kidney samples isolated from transgenic zebrafish (Fig. 1F). Urine collected from 5 dpf larvae did not show significant NanoLuc luciferase activity, confirming the complete reabsorption of vdbp-NanoLuc by PT cells under normal conditions (Fig. 1F,G). These data indicate that the vdbp-NanoLuc protein produced in the liver of *½vdbp-NanoLuc* zebrafish is released into the bloodstream, filtered and reabsorbed by kidney PT cells – so that it is absent from the urine, much like natural VDBP under physiological conditions (Fig. 1G).

### Validation of the bioluminescent vdbp-NanoLuc reporter system in genetic models

**Fig. 2.**
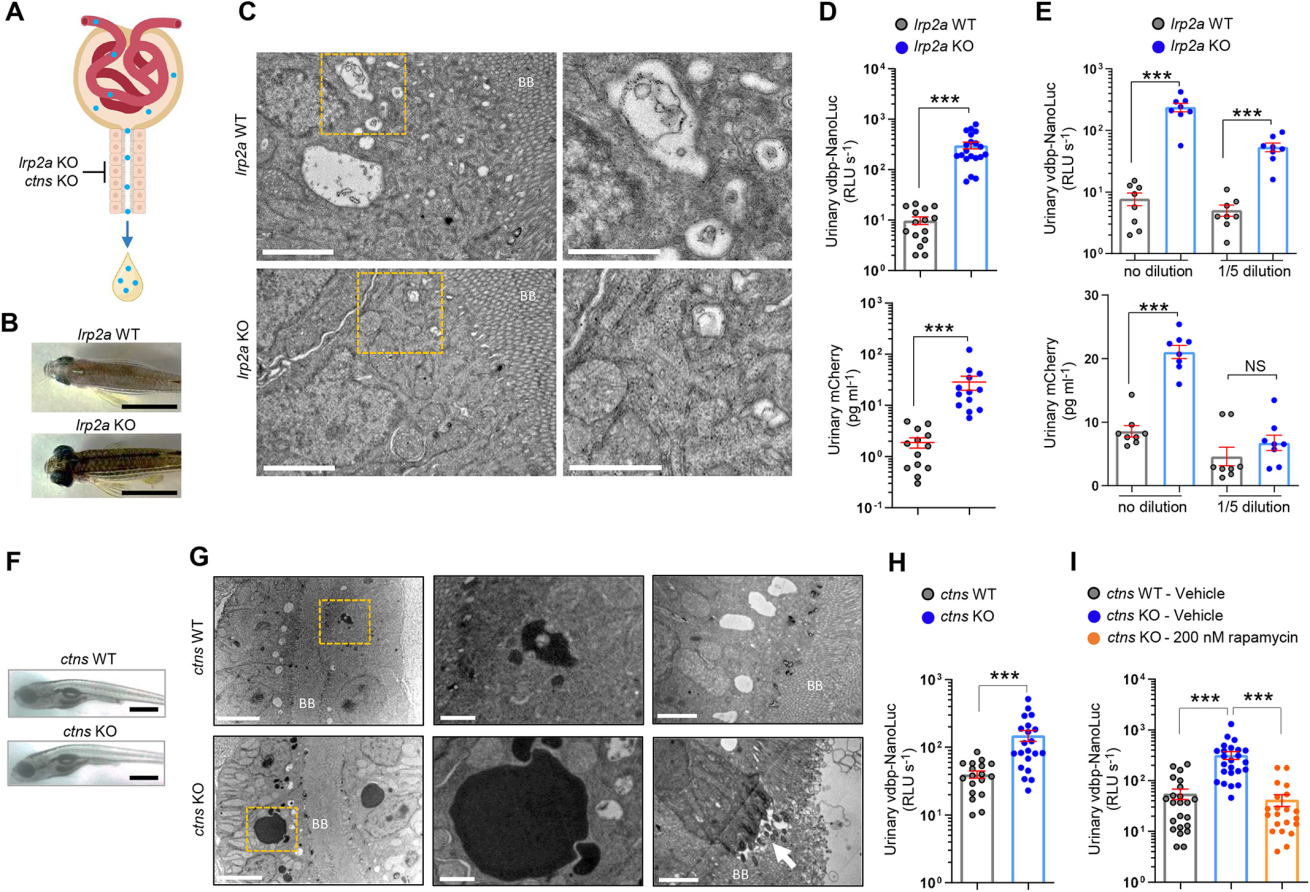
**Validation of the bioluminescent vdbp-NanoLuc reporter system in genetic models.** (A) Schematic illustration of genetic models of PT dysfunction caused by inactivation of megalin receptor [*lrp2a* knockout (KO)] or deficiency of lysosomal cystine transporter cystinosin (*ctns* KO). (B) Dorsal view showing enlarged eye globes in *lrp2a* KO adult zebrafish. Scale bars: 1 cm. (C) Transmission electron microscopy illustrating the loss of endocytic vesicles in the PT of *lrp2a* KO zebrafish larvae. Yellow dashed line squares contain images at higher magnification. *n*=5. BB, brush border. Scale bars: 2 µm (left column), 1 µm (right column). (D) Overnight urine was collected from *lrp2a* WT and *lrp2a* KO *½vdbp-NanoLuc* or *½vdbp-mCherry* zebrafish larvae at 5 dpf, and analyzed by luminometric assay or mCherry enzyme-linked immunosorbent assay (ELISA). *½vdbp-NanoLuc*, *n*=15 (*lrp2a* WT) and *n*=20 (*lrp2a* KO); *½vdbp-mCherry*: *n*=13. (E) Transgenic *lrp2a* mutant *½vdbp-NanoLuc* zebrafish were crossed with *lrp2a mutant ½vdbp-mCherry* zebrafish to produce double-transgenic *lrp2a* KO larvae expressing both VDBP tracers. Urine samples were collected and analyzed without dilution (no dilution) or after 5× dilution in E3 medium (1/5 dilution) for both VDBP reporter proteins in the same urine sample. After 5× dilution, the urinary NanoLuc luciferase activity in *lrp2a* KO larvae was still significantly higher than that in *lrp2a* WT larvae, whereas no difference was observed between the two groups when urine samples were analyzed by mCherry ELISA. *n*=8. (F) Morphology of *ctns* KO larvae at 14 dpf. Scale bars: 1 mm. (G) Representative micrographs showing electron-dense vesicles and sporadic loss of brush border in the PT epithelial cells of *ctns* KO larvae at 14 dpf. *n*=5. Yellow dashed line squares contain images at a higher magnification. Arrow indicates loss of brush border. Scale bars: 5 µm (left column), 1 µm (middle column) and 2 µm (right column). (H) Quantification of vdbp-NanoLuc luciferase activity in urine samples obtained from 14 dpf *ctns* WT and *ctns* KO *½vdbp-NanoLuc* zebrafish larvae. *n*=18 (*ctns* WT) and *n*=21 (*ctns* KO). Urinary vdbp-NanoLuc was slightly increased in transgenic *ctns* WT larvae at baseline when kept in fish facility water and under feeding. (I) *ctns* KO *½vdbp-NanoLuc* larvae were treated with fish facility water containing vehicle or 200 nM rapamycin, followed by assessment of urinary vdbp-NanoLuc at 14 dpf. *n*=23 (*ctns* WT-vehicle), *n*=24 (*ctns* KO-vehicle) and *n*=21 (*ctns* KO-rapamycin). Plotted data represent mean±s.e.m. Nonparametric Mann–Whitney test, ***P<0.001 relative to *lrp2a* WT or *ctns* WT. NS, non-significant.

We validated the vdbp-NanoLuc reporter system in two paradigmatic endolysosomal disorders that induce PT dysfunction and LMW proteinuria (Fig. 2A). We first analyzed *½vdbp-NanoLuc* zebrafish with knockout (KO) of the *lrp2a* gene, which encodes megalin (Saito et al., 1994). The *lrp2a* KO zebrafish have enlarged eye globes in adult fish (Fig. 2B), mimicking the ocular anomalies encountered in Donnai–Barrow syndrome due to *LRP2* variants (Kantarci et al., 2007). The defective endocytosis in *lrp2a*-deficient zebrafish is reflected by a highly reduced number of endocytic vesicles in the subapical compartment of PT cells (Fig. 2C). Quantification of urinary vdbp-NanoLuc at 5 dpf revealed a major, 30-fold increase in bioluminescent VDBP signal in *lrp2a* KO compared to *lrp2a* wild-type (WT) larvae [306±56 versus 9.8±1.8 relative light units (RLU) s^−1^, respectively; *P*<0.001] (Fig. 2D, top). Only a 15-fold increase in VDBP signal was observed in *lrp2a* KO *½vdbp-mCherry* larvae compared to *lrp2a* WT larvae when evaluated by mCherry ELISA (28.4±8.7 versus 1.9±0.4 pg ml^−1^, respectively; *P*<0.001) (Fig. 2D, bottom). The bioluminescence assay thus yielded a 2-fold larger difference in VDBP signal between *lrp2a* KO and WT samples than did ELISA, confirming its enhanced sensitivity (Fig. 2E).

Next, we analyzed the bioluminescent VDBP signal in *ctns*-deficient *½vdbp-NanoLuc* zebrafish larvae (*ctns* KO). Inactivating variants of *CTNS*, which encodes the lysosomal cystine transporter cystinosin, cause cystinosis – a lysosomal storage disorder causing PT dysfunction early in life (Cherqui, 2014). We previously demonstrated that *ctns* KO zebrafish larvae develop cystine accumulation, impairment of lysosomal degradation activity and autophagy, as well as LMW proteinuria (Berquez et al., 2023; Festa et al., 2018). No obvious morphological change was observed in *ctns* KO, compared to *ctns* WT, larvae (Fig. 2F). However, ultrastructural analysis evidenced accumulation of large electron-dense vesicles and sporadic loss of the brush border in PT cells of *ctns* KO larvae (Fig. 2G). A major, 4-fold increase in urinary vdbp-NanoLuc levels was observed in *ctns* KO larvae compared to *ctns* WT *½vdbp-NanoLuc* larvae (150±22 versus 40.1±4.9 RLU s^−1^, respectively; *P*<0.001) (Fig. 2H). Cystine accumulation is known to induce mechanistic target of rapamycin complex 1 (mTORC1) hyperactivation in *ctns*-deficient larvae, which can be rescued by the mTORC1 inhibitor rapamycin (Berquez et al., 2023). Accordingly, we could observe rescue of the abnormal urinary excretion of vdbp-NanoLuc upon treatment with 200 nM rapamycin in *ctns* KO *½vdbp-NanoLuc* larvae, compared to a dimethyl sulfoxide (DMSO)-treated group (42.0±11.1 versus 320±56 RLU s^−1^, respectively; *P*<0.001) (Fig. 2I). These data show that the vdbp-NanoLuc reporter line can reliably and sensitively monitor LMW proteinuria in genetic models of PT dysfunction and is useful for testing therapies.

### The bioluminescent vdbp-NanoLuc reporter for detection of drug nephrotoxicity

**Fig. 3.**
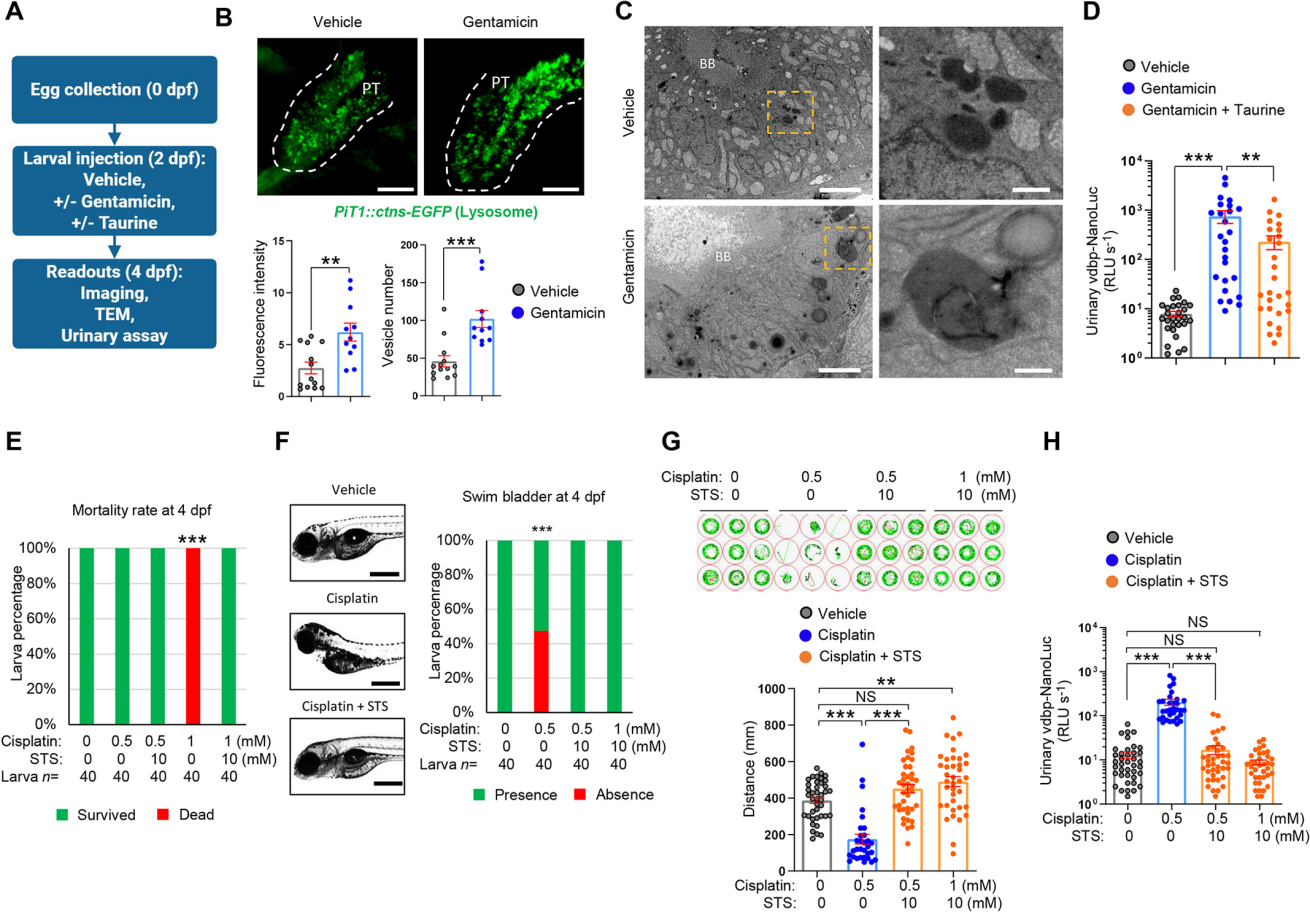
**Bioluminescent vdbp-NanoLuc reporter to detect drug nephrotoxicity.** (A) Experimental protocol used in gentamicin nephrotoxicity assessment and its rescue by taurine co-treatment using transgenic zebrafish larvae. TEM, transmission electron microscopy. (B) Microscopic examination of 4 dpf gentamicin-treated *Tg(PiT1::ctns-EGFP)* zebrafish larvae expressing lysosomal marker Ctns-EGFP in PT cells using multiphoton fluorescence microscopy (top). Accumulation of Ctns-EGFP-positive vesicles is observed in PT cells of gentamicin-treated transgenic zebrafish larvae. Dashed lines outline the proximal tubule. PT, proximal tubule. Relative EGFP fluorescence intensity (bottom left) and EGFP-positive vesicle number (bottom right) are quantified for each proximal tubule. *n*=13 (vehicle), *n*=11 (gentamicin). Scale bars: 30 µm. (C) Ultrastructure of pronephros in 4 dpf larvae treated with gentamicin or vehicle analyzed by transmission electron microscopy. Accumulation of electron-dense vesicles in PT epithelial cells reflects lysosomal phospholipidosis. *n*=5. Yellow dashed line squares contain images at higher magnification. BB, brush border. Scale bars: 5 µm (left column), 1 µm (right column). (D) Urine samples were obtained at 2 days post-injection from *½vdbp-NanoLuc* larvae treated with vehicle, gentamicin alone or gentamicin/taurine and analyzed by luminometry. The variation observed in the gentamicin-injected group may be due to technical limitations of intravenous injection. *n*=28 (vehicle), *n*=26 (gentamicin) and *n*=28 (gentamicin/taurine). (E) Larval lethality in 4 dpf larvae treated with vehicle, cisplatin (0.5 mM or 1 mM) alone or co-incubated with cisplatin (0.5 mM or 1 mM)+10 mM sodium thiosulfate (STS) for 24 h. Treatment with 1 mM cisplatin for 24 h is lethal for zebrafish larvae but rescued by co-treatment with 10 mM STS. *n*=40. (F) Morphological examination of 4 dpf larvae treated with vehicle, 0.5 mM cisplatin alone or co-treatment of cisplatin (0.5 mM or 1 mM)+10 mM STS (left) and semi-quantitative scoring of inflation defects of swim bladder (right). *n*=40. Scale bars: 0.5 mm. (G) Behavioral analysis of 5 dpf zebrafish larvae incubated with vehicle, 0.5 mM cisplatin alone, or co-incubated with cisplatin (0.5 mM or 1 mM)+10 mM STS by movement tracking using the Zebrabox. *n*=40 (vehicle), *n*=31 (cisplatin alone), *n*=40 (0.5 mM cisplatin+10 mM STS), *n*=38 (1 mM cisplatin+10 mM STS). (H) Overnight urine was collected from 5 dpf *½vdbp-NanoLuc* zebrafish larvae treated with vehicle, 0.5 mM cisplatin alone or co-incubated with cisplatin (0.5 mM or 1 mM)+10 mM STS for 24 h and analyzed by luminometry. *n*=39 (vehicle), *n*=31 (0.5 mM cisplatin alone), *n*=39 (0.5 mM cisplatin+10 mM STS), *n*=37 (1 mM cisplatin+10 mM STS). Plotted data represent mean±s.e.m. Nonparametric Mann–Whitney test, **P<0.01 ***P<0.001. NS, non-significant.

To determine whether the vdbp-NanoLuc reporter system can be used for monitoring drug-induced nephrotoxicity, we first exposed *½vdbp-NanoLuc* larvae to gentamicin (Fig. 3A), an aminoglycoside antibiotic causing lysosomal phospholipidosis and PT dysfunction (Chen et al., 2020; Hentschel et al., 2005), as well as structural alterations of the kidney (Bolten et al., 2023). To study lysosome morphology and dynamics in zebrafish larvae, we used the PT-specific reporter line *Tg(PiT1::ctns-EGFP)*, which expresses EGFP-labeled cystinosin (Ctns) (Chen et al., 2020). Exposure to gentamicin induced accumulation of Ctns-EGFP positive vesicles in *Tg(PiT1::ctns-EGFP)* larvae, with increased fluorescent signal intensity (6.2±0.9 versus 2.7±0.6, respectively; *P*<0.01), a higher number of GFP-positive vesicles (102±11 versus 46±7.3, respectively; *P*<0.001) and accumulation of enlarged electron-dense lysosomal compartments in PT cells, compared to the vehicle-treated controls (Fig. 3B,C). These changes were paralleled by a ∼100-fold increase in VDBP signal in gentamicin-treated *½vdbp-NanoLuc* zebrafish larvae compared to that in controls (756±216 versus 7.9±1.0 RLU s^−1^, respectively; *P*<0.001). The PT dysfunction caused by gentamicin toxicity was rescued by co-treatment with taurine (gentamicin+taurine, 230±72 RLU s^−1^; *P*<0.01 versus gentamicin alone; Fig. 3D), as previously described (Chen et al., 2020).

Exposure of PT cells to cisplatin results in cell injury and LMW proteinuria (Wilmes et al., 2015). In rodent models, cisplatin toxicity can be rescued by the addition of sodium thiosulfate (STS) (Dickey et al., 2005). Treatment of *½vdbp-NanoLuc* larvae with 1.5 mM cisplatin for 6 h impaired inflation of the swim bladder (Fig. S2A) and caused abnormal urinary excretion of vdbp-NanoLuc (Fig. S2B). To optimize the protocol for drug screening, we tested lower concentrations of cisplatin for 24 h: incubation of 2 dpf larvae with 0.5 mM cisplatin was non-lethal (Fig. 3E) but induced a 50% incidence of defective swim bladder inflation at 4 dpf (Fig. 3F). The latter resulted in impaired swimming capacity in *½vdbp-NanoLuc* compared to vehicle-treated larvae (175±26 mm versus 387±17 mm, respectively; *P*<0.001) (Fig. 3G). Administration of 10 mM STS rescued lethality (Fig. 3E), as well as swim bladder defects (Fig. 3F) and swimming performance (Fig. 3G), in cisplatin-treated larvae. Exposure of *½vdbp-NanoLuc* larvae to 0.5 mM cisplatin induced a 16-fold increase in the VDBP signal in fish pool water, compared to that in vehicle-treated controls (205±32 versus 12.9±2.2 RLU s^−1^, respectively; *P*<0.001), which was rescued by STS (17.0±3.8 RLU s^−1^; *P*<0.001 versus cisplatin alone) (Fig. 3H). In line, no VDBP signal was detected in larvae co-treated with 1 mM cisplatin and 10 mM STS (Fig. 3H).

Collectively, these data demonstrate that the bioluminescent vdbp-NanoLuc reporter system can reliably monitor nephrotoxin-induced PT dysfunction and identify potential therapies that alleviate drug/chemical nephrotoxicity.

### The vdbp-NanoLuc reporter as biosensor for detecting metal-induced nephrotoxicity

**Fig. 4.**
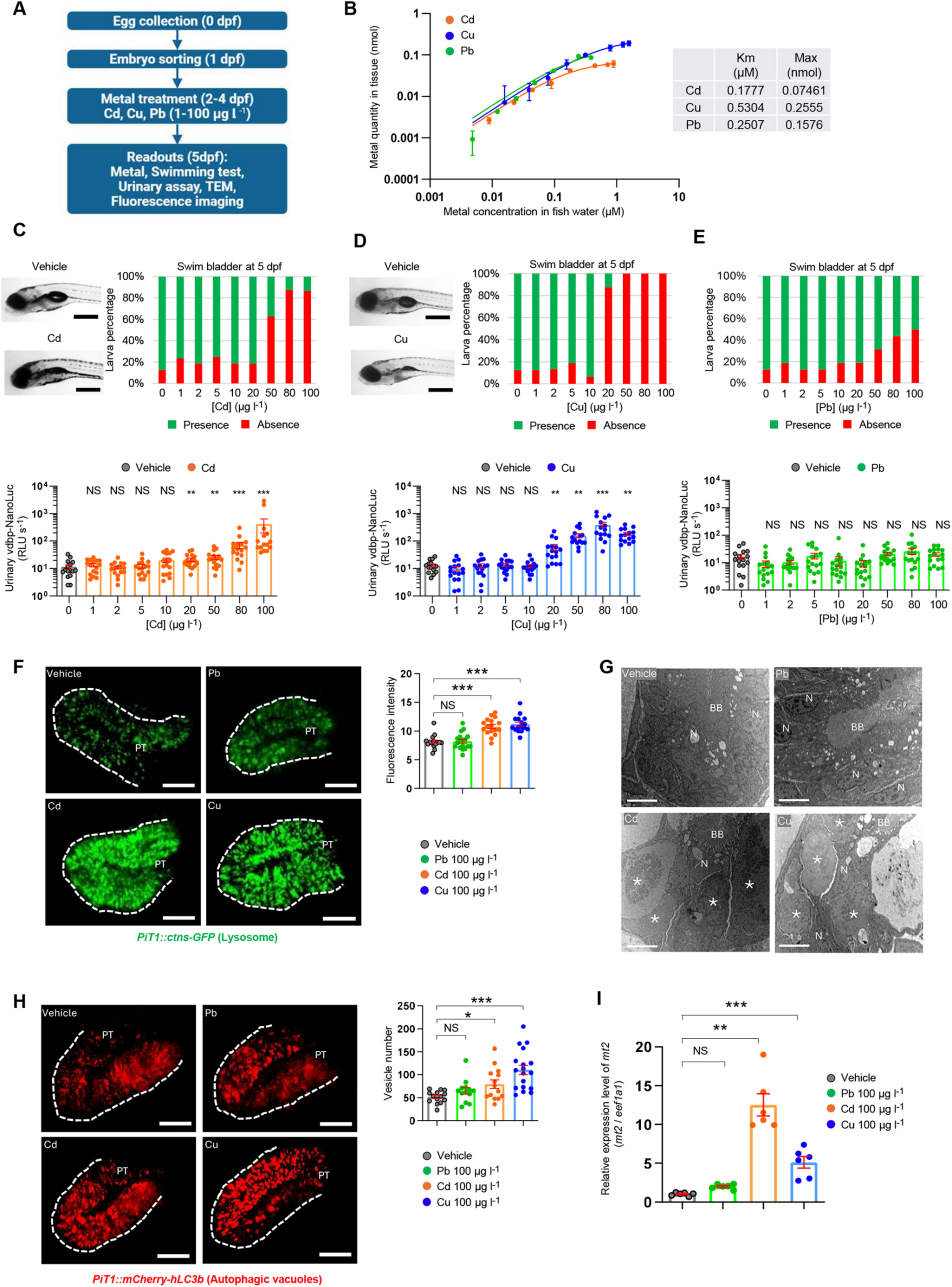
**vdbp-NanoLuc reporter as biosensor for detecting metal nephrotoxicity.** (A) Experimental protocol of metal toxicity assessment using transgenic *½vdbp-NanoLuc* zebrafish larvae. (B) Levels of cadmium (Cd), copper (Cu) and lead (Pb) in larval lysates were analyzed by using inductively coupled plasma mass spectrometry (ICP-MS) after 3 days of larvae incubation with metals. The Michaelis–Menten constant (*K*_m_) and maximal quantity in larvae (Max) are calculated from the measurement by using the molar concentration. The *K*_m_ of Pb is very similar to that of Cd, which is much lower than that of Cu, showing the lower affinity for uptake of Cu by larvae. (C) Morphological examination of 5 dpf larvae treated with vehicle or Cd (top left). Scale bars: 0.5 mm. Semi-quantitative scoring of swim bladder defects and urinary analysis for 5 dpf larvae treated from 2 dpf with Cd at concentrations from 1 to 100 µg l^−1^. Swim bladder inflation is impaired by Cd treatment at 50 µg l^−1^ (top right). Excessive urinary loss of vdbp-NanoLuc using luminometry was observed at 20 µg l^−1^ (bottom). *n*=16. (D) Morphological examination of 5 dpf larvae treated with vehicle or Cu (top left). Scale bars: 0.5 mm. Semi-quantitative scoring of swim bladder defects and urinary analysis for 5 dpf larvae treated with Cu at concentrations from 1 to 100 µg l^−1^. Failure to inflate the swim bladder (top right) and excessive urinary loss of vdbp-NanoLuc (bottom) were observed in larvae treated with Cu from the concentration of 20 µg l^−1^. *n*=16. (E) Semi-quantitative scoring of swim bladder defects and urinary analysis for 5 dpf larvae treated with Pb at concentration from 1 to 100 µg l^−1^. No low-molecular-weight (LMW) proteinuria was seen in any Pb-treated group. *n*=16. (F) Microscopic examination of 5 dpf *Tg(PiT1::ctns-EGFP)* zebrafish larvae expressing lysosomal marker Ctns-EGFP in the PT of larvae treated with vehicle, Pb, Cd or Cu by using multiphoton fluorescence microscopy (left). Accumulation of Ctns-EGFP-positive vesicles in the PT of larvae treated with 100 µg l^−1^ Cd or Cu (right). PT, proximal tubule. *n*=15 (vehicle), *n*=18 (Pb), *n*=17 (Cd), *n*=16 (Cu). Scale bars: 30 µm. (G) The ultrastructure of PT in 5 dpf larvae treated with vehicle, Pb, Cd, and Cu was analyzed by transmission electron microscopy. Accumulation of large electron-dense vesicles corresponding to lysosomes is observed in epithelial cells of larvae treated with 100 µg l^−1^ Cd or Cu. *n*=5. Asterisks indicate large electron dense vesicles. BB, brush border; N, nucleus. Scale bars: 5 µm. (H) Microscopic examination of 5 dpf *Tg(PiT1::mCherry-hLC3b)* zebrafish larvae expressing autophagy marker, mCherry-hLC3b, in PT of larvae treated with vehicle, Pb, Cd or Cu using multiphoton fluorescence microscopy (left). Accumulation of mCherry-hLC3b-positive autophagic vacuoles is observed in PT of larvae treated with 100 µg l^−1^ Cd or Cu (right). *n*=14 (vehicle), *n*=13 (Pb), *n*=14 (Cd), *n*=18 (Cu); Scale bars: 30 µm. (I) Analysis of metallothionein *mt2* mRNA expression levels in metal-treated larvae by quantitative PCR. Total mRNA was extracted from larvae treated with vehicle or 100 µg l^−1^ Pb, Cd or Cu. After reverse transcription, the cDNA was analyzed by quantitative PCR to assess *mt2* mRNA expression levels. *n*=6. Plotted data represent mean±s.e.m. Nonparametric Mann–Whitney test, *P<0.05, **P<0.01, ***P<0.001. NS, non-significant.

The ability of PT cells to reabsorb and concentrate metals such as Cd, Cu and Pb makes the kidney a primary target for metal-induced toxicity. To test the relevance of the vdbp-NanoLuc system in this context, we exposed zebrafish larvae from 2 dpf to environmentally relevant concentrations of Cd, Cu and Pb from 1 to 100 µg l^−1^, and analyzed 5 dpf larvae for swim bladder inflation, swimming behavior, urinary vdbp-NanoLuc, kidney ultrastructure and expression of fluorescent reporter proteins (Fig. 4A).

The uptake of metal contaminants was monitored by inductively coupled plasma mass spectrometry (ICP-MS) analysis of larval lysate, yielding Michaelis–Menten kinetics (Fig. 4B). The uptake kinetic for the three cations were relatively identical, with Michaelis–Menten constant (*K*_m_) ranging from 0.2 µM (Cd and Pb) to 0.5 µM (Cu). Exposure to 20 µg l^−1^ Cd led to significantly higher urinary vdbp-NanoLuc levels, whereas exposure to 50-100 µg l^−1^ Cd impaired swim bladder inflation, in zebrafish larvae (Fig. 4C). Similarly, treatment of larvae with 20 to 100 µg l^−1^ of Cu led to increased urinary vdbp-NanoLuc and swim bladder defects (Fig. 4D). In contrast, Pb exposure led to no detectable increase in urinary vdbp-NanoLuc levels and much milder anomalies in swim bladder inflation (Fig. 4E). The total swimming distance was not affected by treatment with Cd and Cu (Fig. S3A,B) but significantly declined in groups exposed to 20 to 100 µg l^−1^ Pb (Fig. S3C). The improved sensitivity of the vdbp-NanoLuc reporter system, compared to that of the vdbp-mCherry system, was verified for the Cu toxicity model (Fig. S4).

Previous work has shown that Cd exposure impairs lysosome function *in vitro* (Fotakis et al., 2005) and that lysosomes play an important role in Cu homeostasis (Polishchuk and Polishchuk, 2016). To further investigate the mechanism by which metal contaminants yield differential PT toxicity, we assessed lysosome morphology and function using the PT-specific lysosome reporter *Tg(PiT1::ctns-EGFP)* line and autophagy reporter *Tg(PiT1::mCherry-hLC3b*) line (Berquez et al., 2023). Treatment with Cd and Cu induced the accumulation of Ctns-EGFP-positive lysosomes in PT cells [fluorescence intensity, 10.8±0.4 (Cd) versus 11.2±0.4 (Cu) versus 8.2±0.3 (Pb) versus 8.1±0.3 (control); *P*<0.001; Fig. 4F], also reflected by accumulation of enlarged electron-dense vesicles (Fig. 4G). These changes induced by Cd and Cu were paralleled by the accumulation of mCherry-hLC3b-positive autophagic vacuoles in PT cells, compared to Pb- or vehicle-treated larvae, substantiating defective lysosomal processing (Fig. 4H). The expression of the metallothionein Mt2, a 7 kDa cysteine-rich metal-binding protein, can be induced by Cd in zebrafish (Wu et al., 2008). Exposure to metals induced a 12-fold (Cd) and 5-fold (Cu) increase in expression of *mt2* mRNA in *½vdbp-NanoLuc* larvae, whereas no significant change was observed in the Pb-treated group, compared to vehicle-treated controls (Fig. 4I).

### Inter- and intra-assay variability of the VDBP luminometric assay

By analyzing urine samples collected from *lrp2a* KO or gentamicin-treated larvae, the luminometric assay showed low intra-assay variability, with a coefficient of variation (CV) of 3.4% (*lrp2a* KO) and 3.0% (gentamicin) (Fig. S5A-C). The inter-assay CV was 9.8% (*lrp2a* KO) and 8.2% (gentamicin) (Fig. S5A-C). The high intra-assay repeatability and inter-assay reproducibility confirmed the suitability of the vdbp-NanoLuc reporter system for drug screening using genetic and toxic models.

### Use of the vdbp-NanoLuc reporter system for nephron-protective drug screening

**Fig. 5.**
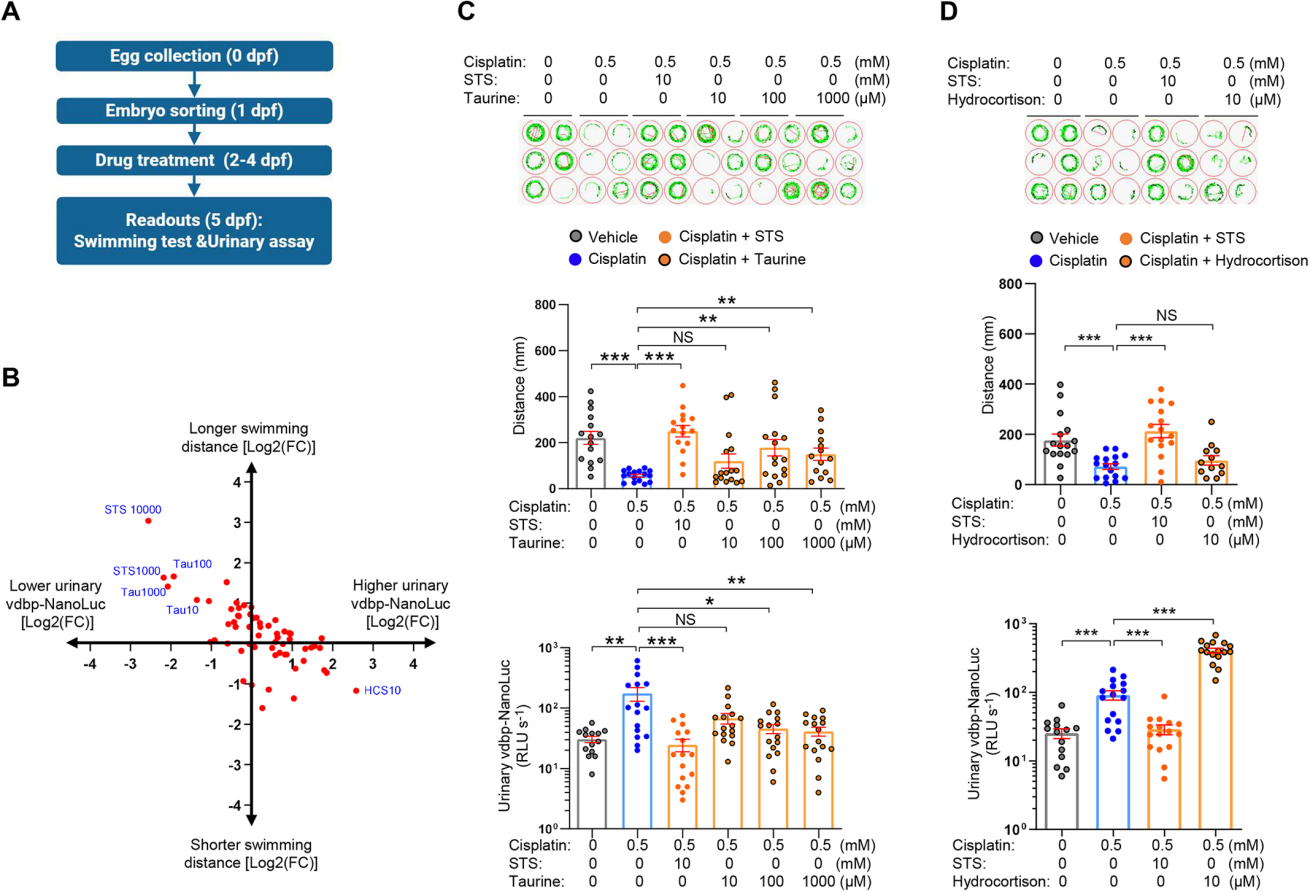
**Use of the vdbp-NanoLuc reporter system for nephroprotective drug screening.** (A) Experimental protocol used in drug screening with transgenic *½vdbp-NanoLuc* larvae to identify antioxidants able to rescue the cisplatin nephrotoxicity. (B) Graphical overview of screening data showing the log2-fold change (FC) in urinary vdbp-NanoLuc, reflecting LMW proteinuria (*x*-axis) and swimming distance (*y* -axis). STS at 10 mM (STS10000) completely rescues cisplatin toxicity as evidenced by restoration of swimming distance and reduction in urinary vdbp-NanoLuc luciferase activity. Urinary vdbp-NanoLuc luciferase activity was partially rescued by co-treatment with 100 µM and 1000 µM taurine (Tau100 and Tau1000, respectively), but luciferase activity was increased with co-incubation with 10 µM hydrocortisone (HCS). Blue, drug name and concentration (µM). (C) Behavioral analysis and urinary analysis for 5 dpf zebrafish larvae incubated with vehicle, cisplatin alone, or co-incubated with cisplatin and 10 mM STS or taurine at different concentrations for 24 h. *n*=16. Overnight urine was collected and analyzed using luminometry. *n*=16. (D) Behavioral analysis and urinary analysis for larvae incubated with vehicle, cisplatin alone, or co-incubated with cisplatin and 10 mM STS or 10 µM HCS for 24 h. Swimming capacity was not further inhibited by HCS co-treatment; however, urinary vdbp-NanoLuc luciferase activity was highly increased by HCS co-treatment. *n*=16. Plotted data represent mean±s.e.m. Nonparametric Mann–Whitney test, *P<0.05, **P<0.01, ***P<0.001. NS, non-significant.

We finally tested the value of the vdbp-NanoLuc system, combined with an automated assessment of larval swimming behavior, to screen for potential compounds able to counteract cisplatin nephrotoxicity (Fig. 5A; Fig. S6). As oxidative stress is involved (Soni et al., 2018), we tested a library of 30 antioxidant compounds including U.S. Food and Drug Administration (FDA)-approved drugs, natural molecules or molecules classified as ‘generally recognized as safe’ (Table S1). To determine non-lethal concentrations, *½vdbp-NanoLuc* larvae were treated from 2 to 5 dpf at a maximal concentration of 1000 µM for water-soluble compounds and 10-100 µM for water-insoluble compounds. Non-lethal concentrations were then used to screen for the rescue of cisplatin toxicity.

Using the combined phenotype analysis, 10 mM STS was able to completely rescue the LMW proteinuria and swimming deficits caused by cisplatin alone, followed by taurine at both 100 µM and 1000 µM (Fig. 5B). Conversely, treatment with 10 µM hydrocortisone increased the urinary level of vdbp-NanoLuc (Fig. 5B). These protective and deleterious effects were confirmed by co-treatment of the *½vdbp-NanoLuc* larvae with STS, taurine or hydrocortisone. Both STS (10 mM) and taurine (100 µM and 1 mM) significantly rescued swimming distance and urinary vdbp-NanoLuc levels (Fig. 5C). Addition of hydrocortisone to cisplatin significantly increased urinary vdbp-NanoLuc levels, compared to cisplatin alone (407±34 versus 91.3±14 RLU s^−1^, respectively; *P*<0.01) (Fig. 5D). Altogether, these data demonstrate the value of the *½vdbp-NanoLuc* larvae for drug screens by combining the bioluminescent urinary assay and swimming behavior analysis.

## DISCUSSION

Here, we establish a bioluminescent LMW proteinuria reporter line based on transgenic zebrafish expressing a 43 kDa vdbp-NanoLuc protein in the liver. The vdbp-NanoLuc system detected LMW proteinuria (abnormal urinary excretion of vdbp-NanoLuc) associated with genetic deletion of endocytic receptor *lrp2a* or lysosomal cystine transporter *ctns*, or by exposure to nephrotoxic drugs and metals. The reporter line detected the effect of small compounds on rescuing LMW proteinuria in genetic disorders and nephrotoxic drug exposure and can be used to identify nephroprotective compounds.

The zebrafish has emerged as a prominent model for studying kidney disease and nephrotoxicity (Outtandy et al., 2018; Poureetezadi and Wingert, 2016). We previously established and validated a vdbp-mCherry reporter zebrafish line for monitoring LMW proteinuria and lysosomal processing in PT cells (Chen et al., 2020). The advantages of the vdbp-NanoLuc system include a 20-fold reduction in cost and 6-fold reduction in processing time for a 96-well plate (Table S2), as well as greater sensitivity and lower intra-assay and inter-assay variability, compared to the vdbp-mCherry system.

A previous NanoLuc-based proteinuria reporter line was developed using the D3 domain of the receptor-associated protein (RAP) (Naylor et al., 2022). RAP is an endoplasmic reticulum-resident chaperone that binds to members of the LDL receptor family including megalin. In PT cells, RAP binds megalin in the endoplasmic reticulum and promotes its proper folding, intracellular trafficking and expression in the apical membrane (Birn et al., 2000). The role of RAP is, thus, mechanistically different from that of VDBP, a bona fide circulating, extracellular endocytic ligand to megalin in PT cells (Nykjaer et al., 1999). Furthermore, recombinant RAP acts as a high-affinity blocker of megalin, interfering with ligand binding and being internalized and dissociated in late endosomes (Czekay et al., 1997). *In vitro*, RAP impairs megalin-mediated endocytic uptake of albumin and fluid-phase markers in opossum kidney cells (Long et al., 2023). These properties indicate that the luminal NanoLuc-D3, derived from RAP, may interfere with the complex process of ligand binding and release by megalin (Beenken et al., 2023; Keller et al., 2024). Of note, three larvae per well had to be used for the NanoLuc-D3 system (Naylor et al., 2022), whereas a single larva per well was sufficient for the *½vdbp-NanoLuc* zebrafish, suggesting that the latter system shows higher sensitivity for detecting PT dysfunction.

The clinical use of cisplatin in chemotherapy is limited by the occurrence of acute kidney injury in 20-30% of patients (Miller et al., 2010). Cisplatin nephrotoxicity has been evidenced in zebrafish, including morphological alterations of PT cells and LMW proteinuria (Chen et al., 2020; Hentschel et al., 2005). STS is the sole FDA-approved medication used to treat cisplatin-induced ototoxicity. In rodents, STS protects cisplatin-treated animals from hearing impairment while reducing kidney dysfunction (Dickey et al., 2005). The protective effect of STS has been attributed to reducing the excessive generation of reactive oxygen species in cochlea (Sheth et al., 2017), which could also play a protective role in kidney PT cells (Festa et al., 2018; Kandel et al., 2025). Our data showing that treatment with antioxidant compounds STS and taurine restores swim bladder inflation and rescues LMW proteinuria in zebrafish support this potential target for cisplatin-induced nephrotoxicity.

Water contamination by metals is a critical environmental issue (Lentini et al., 2017; Rawee et al., 2023). The presence of transporters and endocytic receptors facilitating the uptake of metals explains why the kidney PT is a prime target of metal-induced toxicity (Rawee et al., 2023). Exposure of *½vdbp-NanoLuc* zebrafish larvae to environmentally relevant concentrations of Cd, Cu or Pb yielded specific toxicity patterns. Exposure of larvae to Cd and Cu impaired the inflation of the swim bladder and induced LMW proteinuria, contrasting with the very mild effects of Pb exposure. The LMW proteinuria related to Cd and Cu exposure is associated with accumulation of lysosomes and impaired autophagy in PT cells, a major cause of PT dysfunction (Luciani and Devuyst, 2024), and with increased expression of *mt2* mRNA, in *½vdbp-NanoLuc* larvae. MT2 is a cystein-rich protein known to bind and detoxify heavy metals (Sabolić et al., 2010). Previous work suggests that Cd/MT2 complex is released from intoxicated hepatocytes, filtered and then endocytosed via megalin in PT cells, to be degraded in lysosomes (Nordberg and Nordberg, 2022; Sabolić et al., 2010). Our finding of upregulated Mt2 in zebrafish exposed to Cd is in line with a role of Mt2 in the adaptive response to maintain survival (Hu et al., 2022) and with the use of *mt2* promoter to develop a transgenic zebrafish biosensor for heavy metal detection (Herath et al., 2025).

In contrast with Cd and Cu exposure, Pb exposure did not induce PT dysfunction and LMW proteinuria; however, it impacted both larval development and swim behavior. The mechanism by which Pb exposure causes toxicity in zebrafish larvae warrants further investigation. Because mild proteinuria has been detected in Wistar rats administered drinking water containing 1000 µg l^−1^ lead acetate (Missoun et al., 2010), species factors and/or differences in the type of Pb solution administered, dosage or duration could be involved. The effect of Pb exposure on swimming distance could be a sign of neuronal impairment (Dou and Zhang, 2011; Roy et al., 2014), which could also be involved in defective swim bladder inflation. Of note, low-dose Pb exposure impairs early neurodevelopment, associated with cognitive decline in infants (Lee and Freeman, 2014).

We performed a screen for antioxidant compounds against cisplatin nephrotoxicity by analyzing urinary vdbp-NanoLuc and swimming behaviors. Both traits were significantly rescued with taurine, in line with evidence in rodent models that taurine prevents cisplatin cardiotoxicity (Chowdhury et al., 2016) and brain injury (Owoeye et al., 2018). These results support the use of zebrafish reporter lines for small-molecule screens against nephrotoxicity parameters. Potential limitations of the reporter system lie in the type of promoter used, the tissue specificity, the level of transgene expression, with potential for developmental toxicity, and the stability and biodistribution of the bioluminescent protein. Possible interactions between the drugs tested and NanoLuc luciferase activity (Walker et al., 2017) require validation in a second screening system.

Collectively, these studies demonstrate the value of the *½vdbp-NanoLuc* zebrafish system for congenital and acquired dysfunction of the kidney proximal tubule. The system will be useful to evaluate specific tubular dysfunction associated with other types of kidney disorders and to perform drug screening. This new reporter line further illustrates the value of nanotechnology applied to zebrafish in various disease and toxicology contexts (Cascallar et al., 2022; Choe et al., 2021; Dunuweera et al., 2024; Hason et al., 2022; Herath et al., 2025; Syed and Anderson, 2021; Thierer et al., 2019).

## MATERIALS AND METHODS

### Zebrafish husbandry

Zebrafish (*Danio rerio*) were kept in a day/night cycle of 14/10 h at 28°C in the Fish Facility at the University of Zurich (Zurich, Switzerland). Zebrafish embryos were obtained through natural spawning and raised in zebrafish E3 embryo medium containing 0.01% Methylene Blue. Larvae were fed ZebraFeed 100-200 (Sparos, Portugal) beginning at 5 dpf. The experiments performed on animals were approved by the local legal authority (ZH139/2022**,** Veterinary Office, Canton of Zurich, Switzerland).

### Generation of transgenic *Tg(lfabp::½vdbp-NanoLuc)* zebrafish

Multisite Gateway cloning technology was used to generate the expression vector *lfabp::½vdbp-NanoLuc/cmlc2::EGFP* using plasmids from Tol2 kit v1.2. The mCherry sequence in *pED-½vdbp-mCherry* (Chen et al., 2020) was replaced by NanoLuc luciferase cDNA (Promega, N1001) using BamHI and AscI restriction sites to create a middle-entry clone *pED-½vdbp-NanoLuc*. The vector construction of *lfabp::½vdbp-NanoLuc/cmlc2::EGFP* was generated by LR reaction using the destination vector *pDestTol2CG2* with *cmlc2:EGFP* transgenesis marker, laboratory-generated 5'-entry clone *p5E-lfabp*, middle-entry clone *pED-½vdbp-NanoLuc* and 3′-entry clone *p3E-polyA*, as well as Gateway™ LR Clonase™ II Enzyme Mix (Invitrogen, 11791-020, USA). Plasmid DNA at the concentration of 25 ng µl^−1^ was co-injected with Tol2 transposase mRNA at 50 ng µl^−1^ into WT zebrafish embryos at the one-cell stage to generate mosaic fish. Stable transgenic lines were generated by outcrossing founder zebrafish to AB wild-type fish. Primers used to clone NanoLuc cDNA were as follows: *NanoLuc*-Fw, 5′-ACTGTTGGTAAAGGATCCCCGGTCTTCACACTCGAAGATTTC-3′; *NanoLuc*-Rev, 5′-TGCTCGAAGCGGCGCGCCGCCCCGACTCTAGA-3′. Double-transgenic zebrafish larvae were produced by crossing transgenic *½vdbp-mCherry* zebrafish with transgenic *½vdbp-NanoLuc* zebrafish. For the analysis of mRNA expression level, full-length *½vdbp-NanoLuc* cDNA was amplified using the following primers: *vdbp-NanoLuc-*Fw, 5′-ATGAATGCATCTTTAATTTTAATTTATGCTTTAATAGT-3′; *vdbp-NanoLuc-*Rev, 5′-TTACGCCAGAATGCGTTCGC-3′.

### Drug treatment

For experiments with *lrp2a* and *ctns* lines, KO larvae were compared with WT clutch mates or KO larvae treated with therapeutic molecules. Larvae treated with chemicals (cisplatin or gentamicin) alone were compared with larvae co-treated with chemical and therapeutic molecules. The number of larvae per group is indicated in the legend for each experiment. All larvae were randomly assigned to groups to avoid bias.

For rescue of LMW proteinuria by rapamycin, *ctns* zebrafish larvae were incubated in fish facility water containing 200 nM rapamycin (MedChemExpress, HY-10219) from 5 to 14 dpf, with daily refreshment of treatment conditions. At 14 dpf, overnight urine samples were collected for biochemical analysis.

For gentamicin treatment, 2 dpf embryos were injected with 1 nl of 6 µg µl^−1^ gentamicin (Sigma-Aldrich, G1264)/0.9% NaCl solution alone or co-injected with 6.3 mg ml^−1^ taurine (Sigma-Aldrich, T0625) into the common cardinal vein after anesthesia with 0.2 mg ml^−1^ ethyl 3-aminobenzoate methanesulfonate salt (MS222; Sigma-Aldrich, E10521). Embryos were immediately returned to fresh E3 medium until urinary analysis at 4 dpf.

The toxicity of cisplatin was assessed after incubating 2 dpf embryos in E3 medium containing 0.5 mM or 1 mM cisplatin (Sigma-Aldrich, C2210000) for 24 h. Cisplatin was dissolved in E3 medium at room temperature with constant stirring for 6 h. The rescue of cisplatin toxicity was performed using 3 mM or 10 mM STS (Sigma-Aldrich, 72049). Treatment of 10 mM STS did not affect viability and pronephros morphology in healthy larvae (Al-Dahmani et al., 2022). A pilot study showed that 10 mM STS was well tolerated by WT larvae over 3 days of consecutive treatment (from 2 to 5 dpf). Larvae were co-treated with cisplatin and STS, followed by incubation in fresh E3 medium containing STS. Larvae treated only with vehicle or cisplatin, followed by incubation in E3 medium, were used as controls. The inflation of the swim bladder at 4 dpf and mortality rate at 5 dpf were assessed under an Olympus stereomicroscope.

### Proof-of-concept drug screening to rescue cisplatin toxicity

The protocol used for drug screening included determination of non-lethal concentration and rescue of cisplatin toxicity. Water-soluble compounds were tested at concentrations of 10-1000 µM, which were directly dissolved in E3 or cisplatin-E3 solutions. As cisplatin is not compatible with DMSO, water-insoluble compounds were first dissolved in N,N-dimethylformamide (Sigma-Aldrich, 227056) and were tested at concentrations of 1-100 µM. All compounds used for drug screening were purchased from Sigma-Aldrich (Table S1). The non-lethal concentration of each chemical was determined by incubation of WT larvae in E3 medium from 2 to 5 dpf with daily refreshment of treatment conditions. Twenty larvae were tested for each condition.

The protocol used to rescue cisplatin toxicity was designed in view of large-scale screens, including automated movement tracking. Drug treatment, urine collection and movement tracking were performed in 96-well microplates. Transgenic *½vdbp-NanoLuc* embryos were sorted at 1 dpf by EGFP fluorescence in heart and dispensed in 96-well microplates with one embryo per well using an EggSorter (Bionomous, Switzerland). At 2 dpf, larvae were treated with 200 µl of 0.5 mM cisplatin alone or supplemented with antioxidant (STS or other drug candidates). Twenty-four hours after cisplatin exposure, 150 µl treatment solution was removed and replaced with 150 µl fresh E3 medium alone or antioxidant-containing E3 medium. The larvae were then incubated for 48 h with daily refreshment of treatment solution. Then, 50 µl urine-containing E3 medium was collected from each well at 5 dpf for analysis by luminometry. The 96-well microplates containing zebrafish larvae and the remaining 150 µl of treatment solution were then used to track fish movement over a 5 min swim trial using a Zebrabox (ViewPoint, France) to evaluate behavior and total swim distance. Larvae co-treated with cisplatin and STS were used as a positive control, and larvae treated with cisplatin alone were used as a negative control, for each microplate. Drug effects were evaluated by comparing the cisplatin and antioxidant co-treated larvae with those treated with cisplatin alone. Data were graphically represented with a log2-fold change in both quantification of NanoLuc luciferase activity/LMW proteinuria (relative light intensity, *x*-axis) or total swimming distance (*y*-axis).

### Metal exposure and analysis by ICP-MS

To conduct exposure to trace metals, plastic consumables were pre-washed with acid and ultrapure baths. Metal solutions used in this study consisted of Cu(NO_3_)_2_, Cd(NO_3_)_2_ and Pb(NO_3_)_2_, all prepared from metal standards (Fluka). Stock solutions (1000 mg l^−1^) were diluted in 1% nitric acid to prepare intermediate (100 mg l^−1^) solutions, which were diluted again in E3 medium to have working solutions containing 100 µg l^−1^ metal. Following pH adjustment with NaOH, working solutions were further diluted in E3 medium to obtain solutions at concentrations ranging from 1 to 80 µg l^−1^. For both treatments, 200 µl metal solution per larva was used in a 96-well plate (with one larva per well, urinary analysis) or a 12-well plate (with ten larvae per well, for metal quantification in larval lysate). Following metal exposure, ten larvae for each group were collected and placed in pre-washed 1.5 ml tubes. After removal of excessive treatment solution, larval tissues were transferred into new 1.5 ml tubes, snap frozen in liquid nitrogen and stored at −80°C until sample processing.

Each frozen larval tissue sample was digested using 100 µl of 65% nitric acid and left at room temperature overnight. Larval samples were diluted with ultrapure water to reach a final concentration of 1% HNO_3_ and a volume of 10 ml. Concentrations of Cd, Cu and Pb were measured by using ICP-MS (7700x, Agilent Technology) under collision mode with He gas to avoid polyatomic interferences (Worms and Slaveykova, 2022). Data obtained were used to investigate Michaelis–Menten uptake kinetic parameters for each metal salt.

### Fluorescence microscopy

A light-sheet fluorescence microscope (Lightsheet Z.1, Zeiss, Germany) was used for *in vivo* visualization of EGFP expression in the hearts of *½vdbp-NanoLuc* zebrafish larvae using a 20×/1.0 NA water immersion objective (W Plan APO, Zeiss).

A Leica SP8 MP DIVE FALCON multiphoton microscope was used for imaging of Ctns-EGFP (lysosome marker) and vdbp-mCherry (endocytic ligand) using an HC IRAPO L 25×/1.0 W motCORR objective lens after excitation by a two-photon laser at 920 nm (EGFP) or 1040 nm (mCherry). Zebrafish larvae bred into the nacre background were used for experimentation and collected after tail removal followed by fixation in 4% paraformaldehyde overnight at 4°C. After three washes with PBS containing 0.1% Tween 20, zebrafish samples were embedded in 1% low-melting-temperature agarose for 3D optical imaging. The images acquired with multiphoton microscopy were processed with Huygens (Scientific Volume Imaging) for deconvolution. Total vesicle number in each pronephric PT was automatically quantified using Imaris software (Bitplane), and total fluorescent intensity was quantified using ImageJ and presented as relative fluorescent intensity.

### Bioluminescence microscopy

Visualization of vdbp-NanoLuc protein in larvae was carried out at the Photonic Bioimaging Center at the University of Geneva. Five days post-fertilization *½vdbp-NanoLuc* larvae were immobilized using MS222 and incubated in E3 medium containing furimazine (1 µl furimazine per 100 µl E3 medium) and observed using an LV200 microscope (Olympus, Germany).

### Transmission electron microscopy

*lrp2a* larvae and *ctns* larvae were collected at 5 or 14 dpf for ultrastructural analysis. The tails of the larvae were removed before overnight fixation in 2.5% glutaraldehyde and 1.6% paraformaldehyde in 0.1 M pH 7.3 cacodylate buffer. Samples were then rinsed three times in 0.1 M cacodylate buffer, post-fixed in 1% osmium tetroxide/cacodylate buffer for 40 min and stained in 1% aqueous uranyl acetate for 1 h. Following dehydration through a graded series of ethanol solutions, larvae were infiltrated overnight in 50% Epoxy embedding medium (Epon812 substitute, Sigma-Aldrich, 45345)/Propylene Oxide (Sigma-Aldrich, 82320), followed by embedding in Epon812 at 60°C for 28 h. Semi-thin sections (350 nm) were prepared using a Leica EM FCS ultra-microtome (Leica Microsystems, Germany), stained with Toluidine Blue and examined under a light microscope. Ultra-thin sections (60 nm) were collected onto formvar-coated copper grids, stained with lead phosphate aqueous solution and visualized using an electron microscope (FEI Tecnai G2 Spirit) at 120 kV. ImageJ software was used to analyze the images.

### Tissue isolation and urine collection

Liver and kidney tissue were extracted from transgenic adult zebrafish in Nano-Glo^®^ Luciferase Assay Buffer (Promega, N1120) and sonicated with a Digital Sonifier (Branson, 102C). Individual overnight urine samples were collected from 5 or 6 dpf larvae in 96-well plates with 100 or 200 μl E3 medium per well, respectively. At 14 dpf, 500 μl fish facility water was used to culture individual larvae for 16 h at 28.5°C to collect the overnight urine for both *½vdbp-mCherry* and *½vdbp-NanoLuc* larvae, and 50 µl fish pool water was collected for ELISA or luminometric analyses. Samples were stored at −20°C until the ELISA analysis, whereas freshly collected fish pool water or tissue lysates were immediately assessed for NanoLuc luciferase activity.

### mCherry ELISA

The mCherry ELISA procedure has been described in our previous study (Chen et al., 2020). Urinary vdbp-mCherry was quantified using an mCherry ELISA kit (Abcam, ab221829), with 50 µl fish pool water mixed with 50 µl solution containing the capture antibody and detector antibody using a 96-well microplate. Samples in each well were then incubated at 37°C for 1 h, rinsed three times with wash buffer PT, and incubated with 100 μl TMB substrate at room temperature for 10 min. The reaction was stopped by adding 100 μl stop solution into each well, and absorbance was read at 450 nm.

### Bioluminescent assay

NanoLuc luciferase activity was assessed in 96F non-treated, white microwell plates (Thermo Fisher Scientific, 236108) for luciferin furimazine (Promega, N1120) and hikarazine Z108 (Synthelis Biotech, France) (Coutant et al., 2020). To prepare the hikarazine Z108 solution, 1 mg of hikarazine Z108 powder was dissolved in 0.2 ml DMSO, followed by the addition of 0.3 ml of acidic ethanol, which is prepared by adding 100 μl of 37% hydrochloric acid in 12 ml of 100% ethanol. The solution was incubated for 2 h in a 50°C water bath then stored at −20°C until the time of assay. A custom-made NanoLuc assay buffer (NAB) was prepared for the urine assay using hikarazine Z108. The NAB buffer consisted of 100 mM MES, pH 6.0, 1 mM CDTA, 0.5% Tergitol NP-40, 0.05% Antifoam 204, 150 mM KCl, 1 mM DTT and 35 mM thiourea (Hall et al., 2012; Xiong et al., 2022). For both furimazine and hikarazine Z108, 50 μl fish pool water or tissue/larval lysate was assayed with 50 μl substrate/buffer mix. The E3 medium/fish facility water incubated alone was used as blank for normalization. The bioluminescence signal was measured using an Infinite M Plex Microplate reader (TECAN, Switzerland) in triplicate over a 10 min span following the addition of the substrate/buffer mix. Light intensity is expressed as RLU s^−1^.

We optimized the bioluminescence assay protocol by evaluating two distinct concentrations for each substrate. Serial dilutions of larval lysates of *½vdbp-NanoLuc* larvae in fish E3 medium were incubated with 0.5 µl or 1 µl furimazine per assay. The light intensity obtained from reactions using 0.5 µl furimazine was similar to values obtained with 1 µl (Fig. S1B). We tested two concentrations of hikarazine Z108: 0.31 µl per assay (final concentration of 13 µM), as suggested by Coutant et al. (2020), or 0.5 µl per assay (final concentration of 21 µM), resulting in higher light intensity with the latter (Fig. S1C). Light intensity was directly proportional to the relative concentration of NanoLuc luciferase for both substrates (Fig. S1B,C). Light signals obtained with hikarazine Z108 were approximately three times greater than those with furimazine (Fig. S1D). Furimazine was used as standard substrate for the studies, except for the screening experiments, for which hikarazine Z108 was used owing to its lower cost.

### Quantitative real-time PCR

Zebrafish larvae were homogenized in 1 ml PureZOL™ RNA Isolation Reagent (Bio-Rad, 732-6880) using T10 Basic Ultra-Turrax Disperser (IKA, Staufen, Germany). Total RNA was extracted and purified by an Aurum Total RNA Fatty and Fibrous Tissue Kit (Bio-Rad, 732-6830). Genomic DNA was removed by on-membrane DNAse I treatment during RNA purification. 1000 ng RNA was converted into cDNA by reverse transcription with iScript cDNA Synthesis Kit (Bio-Rad, 1708890), followed by determination of mRNA expression levels using quantitative PCR with iQ SYBR Green Supermix (Bio-Rad, 1708880) and a CFX96 real-time PCR Detection System (Bio-Rad, 1845097). Quantitative PCR primers were designed using Primer3. The sequences were as follows: zf-eef1a1a-Fwd, 5′-TTCTCCGAGTATCCTCCTCTG-3′; zf-eef1a1a-Rev, 5′-CTTCTCCACTCCTTTAATCACTCC-3′; zf-mt2-Fwd, 5′-CCTCCAGCATCAACTCATTCAC-3′; zf-mt2-Rev, 5′-CAACTCTTCTTGCAGGTAGTACAC-3′. PCR temperature cycling conditions were 95°C for 3 min, followed by 40 cycles of 15 s at 95°C and 30 s at 60°C. Relative changes in mRNA level of target genes over housekeeping gene *eef1a1a* were calculated using the delta-delta Ct method.

### Western blotting

Proteins were extracted from liver isolated from adult zebrafish or larval tissue using RIPA buffer (Sigma-Aldrich, R0278) supplemented with 20% glycerol and after sonication with a Digital Sonifier (Branson, 102C). Protein concentration was determined using Bradford protein assay (Bio-Rad, 5000002). Proteins were separated by SDS-PAGE under reducing conditions and transferred onto PVDF membrane. Non-specific binding sites were blocked by incubation in 5% non-fat milk (Bio-Rad, 1706404) diluted in PBS. The membrane was incubated overnight at 4°C with primary antibody, then peroxidase-labeled secondary antibody (Dako, Denmark). Peroxidase activity was assayed with Immobilon Western Chemiluminescent HRP Substrate (Millipore, WBKLS0500) and visualized with a ChemiDoc™ Touch Imaging System (Bio-Rad). The following antibodies were used in this study: anti-NanoLuc luciferase (Promega, N7000; 1:500), anti-Gapdh (Cell Signaling Technology, 14C10; 1:500), anti-GFP (Thermo Fisher Scientific, A-11122; 1:1000).

### Statistical analysis

Statistical analyses were performed using Prism 5 software (GraphPad Software). A D'Agostino-Pearson normality test was applied on all datasets. Unpaired two tailed *t*-test or Mann–Whitney non-parametric tests were used to compare treatment groups.

Zebrafish embryos were randomly selected for chemical-treatment groups or control groups. Statistical significance was set at a *P*<0.05.

### Use of artificial intelligence tools

No artificial intelligence tools were used in this study, including in writing the manuscript.

## Supplementary Material

10.1242/dmm.052673_sup1 Supplementary information
