# Supplementary material for: A zebrafish luminescent biosensor for kidney tubulopathy, metal toxicity and drug screening
Source: Dis Model Mech. 2026 May 22;19(5):dmm052673. doi: 10.1242/dmm.052673 (PMC13225708; doi:10.1242/dmm.052673)
Supplement: Supplementary information [file dmm-19-052673-s1.pdf]

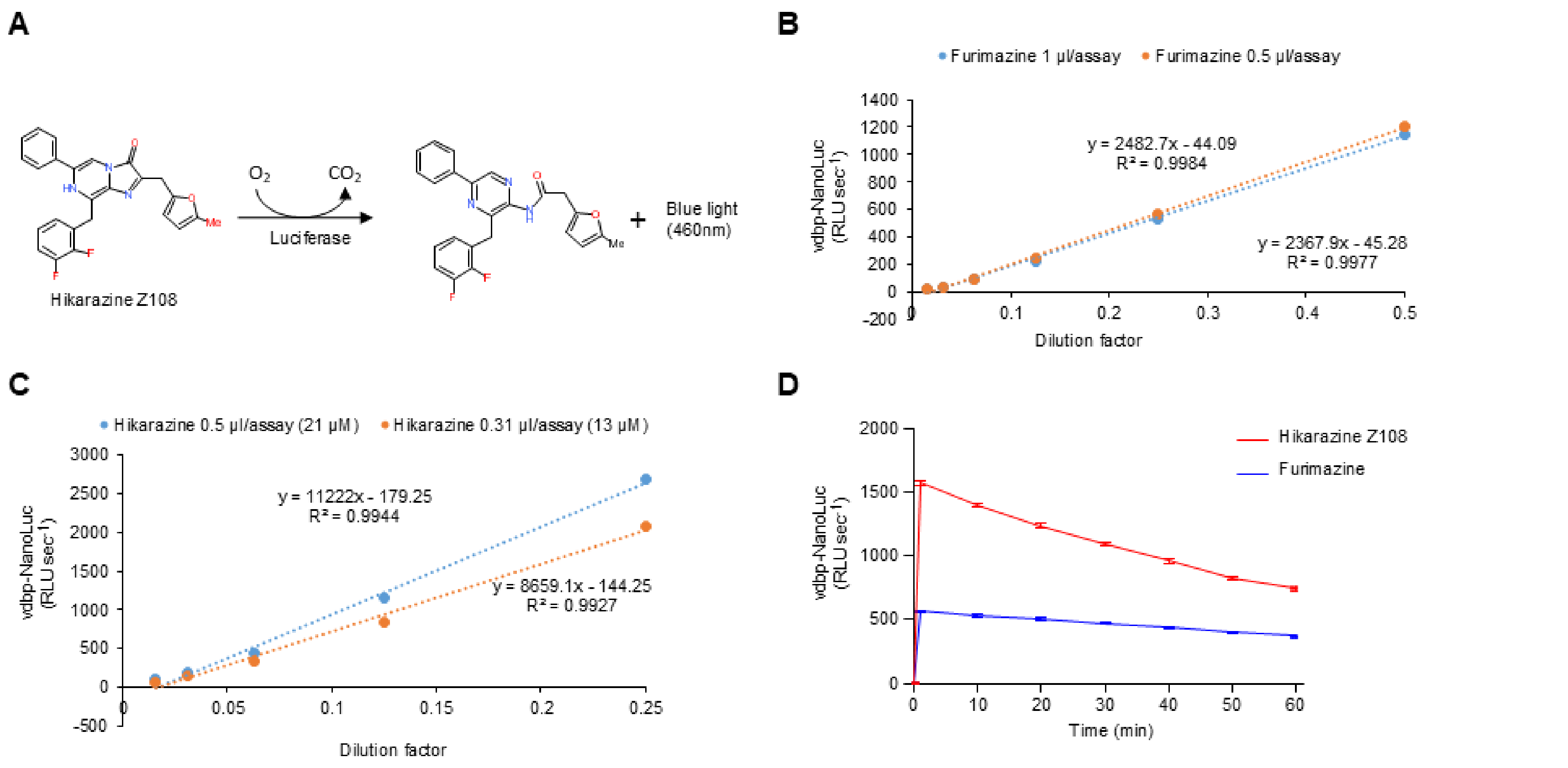

Fig. S1. Luciferins and optimization of NanoLuc luciferase assay.

(A) The oxidation of hikarazine Z108 by NanoLuc luciferase in the presence of O_2 produces blue light at a wavelength of 460 nm. (B) Bioluminescent VDBP signal from NanoLuc luciferase assay performed with a serially diluted larval lysate containing vdbp-NanoLuc and its substrate furimazine. Quantification with 1µL furimazine in a final volume of 100 µL per assay were compared with those using 0.5 µL furimazine. *n*=4 replicates. (C) Bioluminescent VDBP signals quantified from NanoLuc luciferase assay using a serially diluted larval lysate containing vdbp-NanoLuc and hikarazine Z108 as substrate. 0.31 µL hikarazine Z108 in a final volume of 100 µL per assay (13µM) and 0.5 µL in 100 µL per assay (21 µM) were tested. *n*=4 replicates. (D) Dynamic changes of bioluminescent VDBP signal measured at different time points over a period of 60 min. The same amount of larval lysate containing vdbp-NanoLuc were used in reactions for both furimazine and hikarazine Z108. *n*=8 replicates.

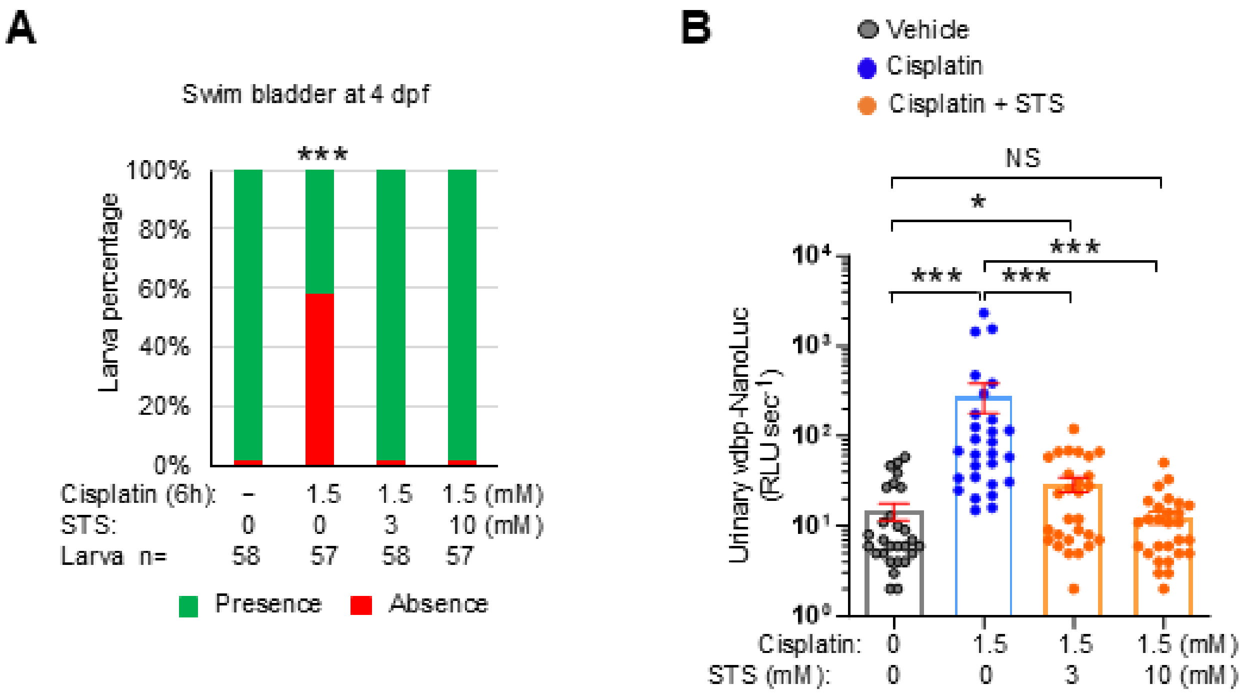

Fig. S2. Rescue of cisplatin toxicity by co-treatment with sodium thiosulfate.

(A) Morphological examination of 4 dpf larvae treated with vehicle, 1.5mM cisplatin alone (6 hours) or co-treated with a mixture of cisplatin + sodium thiosulfate (STS) at 2 dpf for 6 hours by semi-quantitative scoring of developmental defects of swim bladder inflation. Bar = 0.5 mm. (B) Urinary analysis of overnight urine collected from 5 dpf transgenic *½vdbp-nanoLuc* larvae treated with vehicle, 1.5mM cisplatin alone or co-treated with a mixture of cisplatin + STS (3 or 10 mM) at 2 dpf for 6 hours, by luminometry; *n*=28. **P*<0.05, ****P*<0.001; NS, non-significant.

Disease Models & Mechanisms • Supplementary information

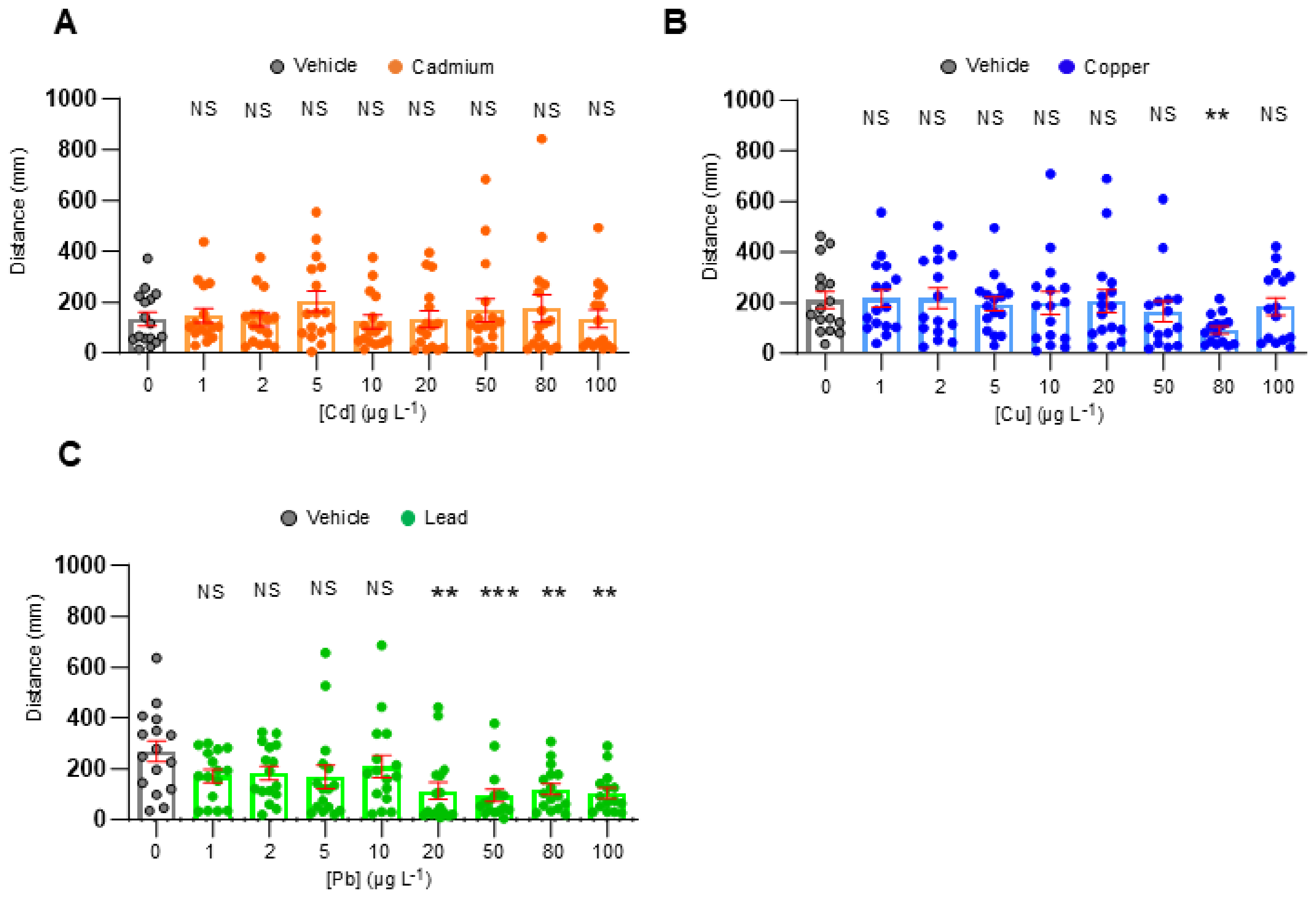

Fig. S3. Assessment of swimming behavior in larvae treated with metals.

(A) Movement tracking using Zebrabox for 5 dpf larvae incubated with vehicle or Cd at concentrations from 1 to 100 µg L^{-1}. (B) Behavioral analysis of 5 dpf larvae incubated with vehicle or Cu at concentrations from 1 to 100 µg L^{-1}. Swimming distance was impaired only in larvae treated with 80 µg L^{-1} Cu. (C) Behavioral analysis of 5 dpf larvae treated with vehicle or Pb at concentrations from 1 to 100 µg L^{-1}. Swimming distance declined significantly in groups treated with 20 to 100 µg $L^{-1}$of Pb. *n*=16. ***P*<0.01, ****P*<0.001; NS, non-significant.

Disease Models & Mechanisms • Supplementary information

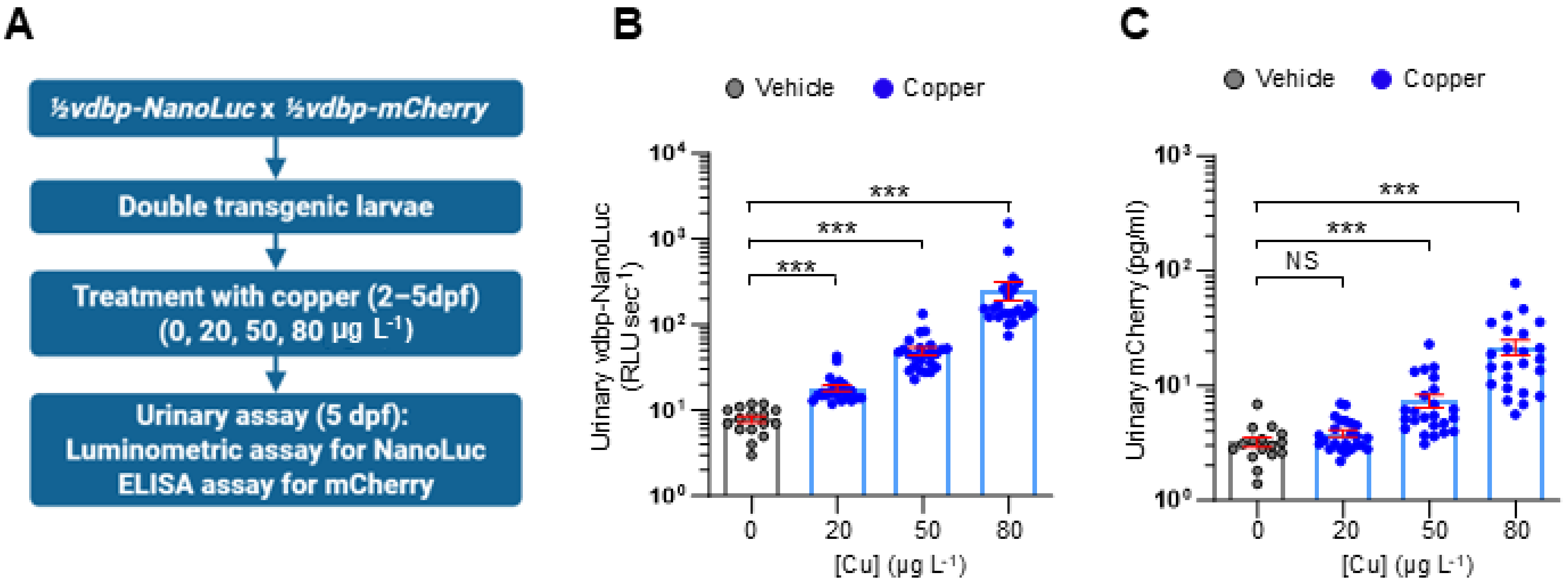

Fig. S4. Comparative studies of LMW proteinuria evaluation using vdbp-NanoLuc and vdbp-mCherry in Cu toxicity models.

(A) Transgenic *½vdbp-NanoLuc* zebrafish were crossed with *½vdbp-mCherry* zebrafish to produce double transgenic larvae expressing both vdbp-NanoLuc and vdbp-mCherry tracers. Transgenic larvae were treated with Cu at concentrations of 0, 20, 50 and 80 µg L^{-1}. Urine samples were collected at 5 dpf and analyzed for both VDBP reporter proteins from the same urine sample. (B) Urinary NanoLuc luciferase activity was quantified by luminometric assay. The bioluminescent signals of all three Cu-treated groups were significantly higher than the vehicle-treated control. *n*=16 (vehicle), *n*=24 (Cu-treated group). (C) The quantity of vdbp-mCherry in the same urine samples assessed in (B) was analyzed by mCherry ELISA. No difference was observed between the 20 µg L^{-1} Cu-treated group and vehicle-treated control. *n*=16 (vehicle), *n*=24 (Cu-treated group). ***P<0.001; NS, non-significant.

Disease Models & Mechanisms • Supplementary information

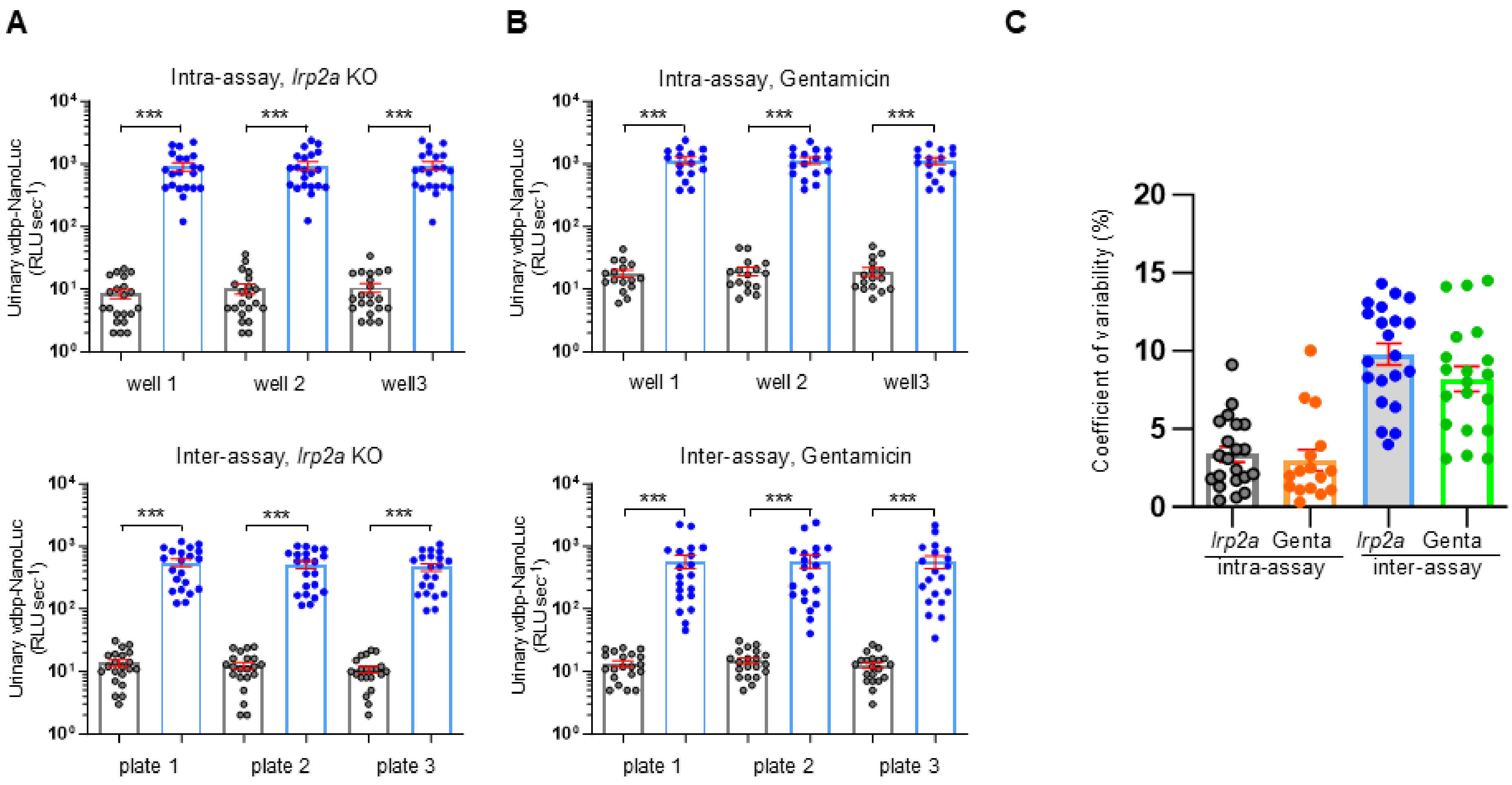

Fig. S5. Inter- and intra-assay variability for quantification of vdbp-NanoLuc by luminometry.

(A) Urine samples collected from *lrp2a KO* larvae were analyzed by luminometry in triplicates (3 wells for each sample in the same microwell plate, intra-assay variability) and in 3 different runs (1 well per plate across 3 plates, inter-assay variability). (B) Urine samples collected from gentamicin-treated larvae were analyzed by luminometry in triplicates (intra-assay variability) and in 3 different runs (inter-assay variability). (C) Calculation of intra- and inter-assay coefficient of variability (%) with data obtained from (A) and (B). ***$P<0.001$.

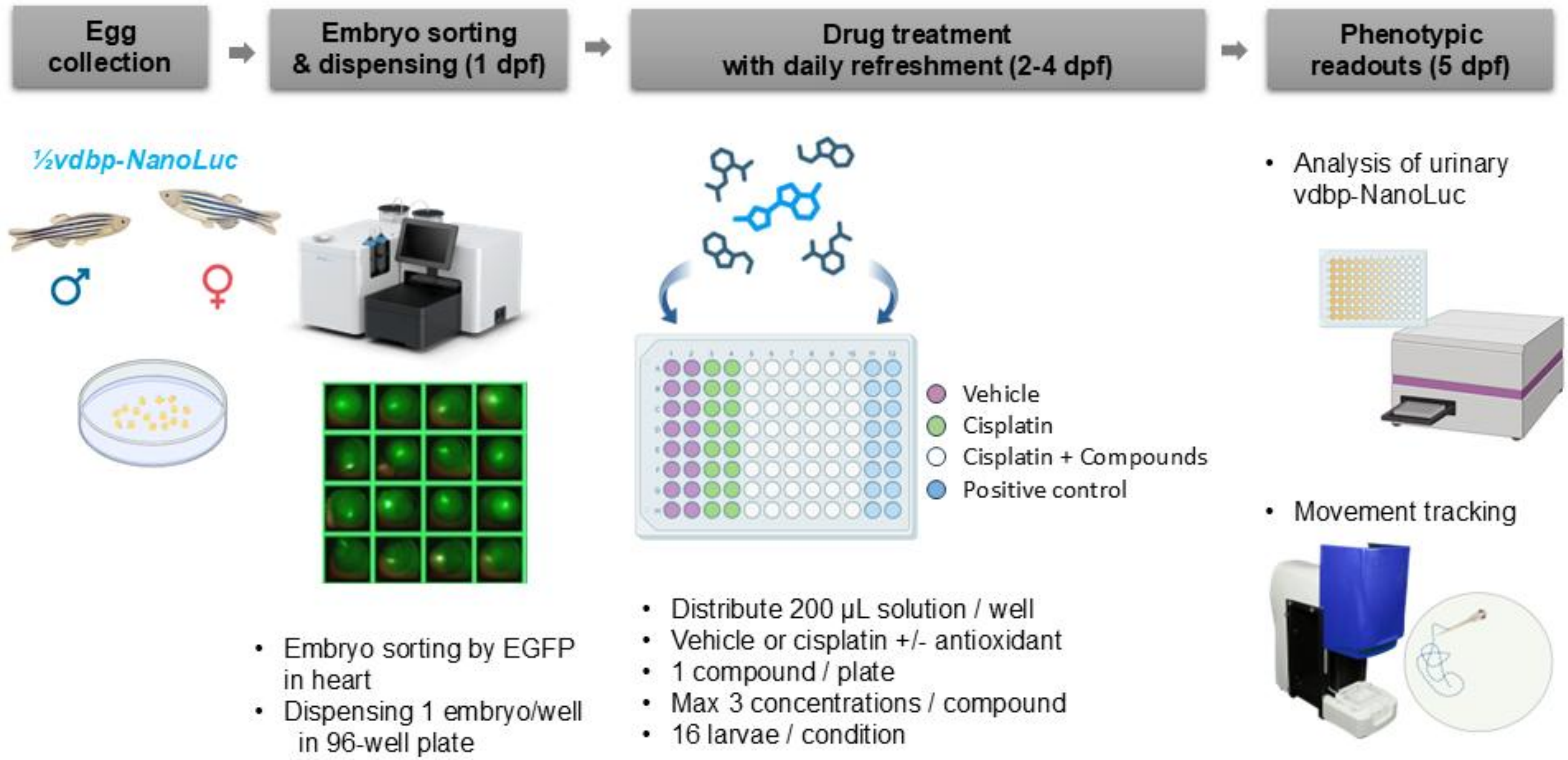

Fig. S6. Experimental protocol used to drug screen antioxidants able to rescue cisplatin-induced nephrotoxicity.

Bioluminescent *½vdbp-NanoLuc* larvae were treated with cisplatin and antioxidants from 2 dpf in 96-well microplates with treatment effects assessed at 5 dpf. At 1 dpf: automated embryo sorting by EGFP fluorescence in heart and dispensing into a 96-well plate; At 2-4 dpf: treatment of larvae with 200 µL cisplatin alone or a mixture of cisplatin + antioxidant solution, with daily refreshment of treatment; At 5 dpf: quantification of urinary vdbp-NanoLuc using 50 µL of urine/fish pool water and movement tracking of larvae in 150 µL of urine/water in a 96-well microplate.

Disease Models & Mechanisms • Supplementary information

Table S1. List of antioxidants used in screening and their non-lethal concentrations determined by treatment in wild-type zebrafish. Water-soluble compounds are tested with a maximal concentration of 1000 µM, while water-insoluble compounds are used at a maximal concentration of 100 µM. * Solubility in DMSO.

Name	Reference		Vehicle	Solubility (mM)	Stock (mM)	1000 µM	100 µM	10 µM	1 µM
Aminothiazole (AMT)	123129	FDA approved	water	1000	10	OK	OK	OK	--
Betaine (BTN)	B2629	FDA approved	water	13419	10	OK	OK	OK	--
Cysteamine (CYTM)	M6500	FDA approved	water	3521	10	OK	OK	OK	--
Gallic Acid (GLA)	G7384	Natural	water	70	10	lethal	OK	OK	--
N-Acetylcysteine (NAC)	A7250	FDA approved	water	613	10	lethal	OK	OK	--
Sodium thiosulfate (STS)	72049	FDA approved	water	4427	10	OK	OK	OK	--
Taurine (Tau)	T8691	GRAS	water	500	10	OK	OK	OK	--
Dimethyl Fumarate (DMFM)	242926	FDA approved	DMF	83.0	50	--	lethal	OK	OK
D-Pinitol (DPNT)	441252	Natural	DMF	25.8	10	--	--	OK	OK
Farnesol (FNS)	F203	GRAS	DMF	90.1	50	--	lethal	OK	OK
Ferulic acid (FRA)	PHR1791	FDA approved	DMF	103.1	100	--	lethal	OK	OK
Flavone (FVN)	F2003	GRAS	DMF	198 *	100	--	lethal	OK	OK
Flavanone (FVNN)	102032	GRAS	DMF	196 *	100	--	lethal	OK	OK
Hesperidin (HSP)	H5254	GRAS	DMF	49.2 *	50	--	OK	OK	OK
4-Hydroxyphenylacetic acid (HPA)	H50004	Natural	water	329	10	--	OK	OK	OK
Hydrocinnamic acid (HCA)	135232	Natural	DMF	200 *	100	--	lethal	OK	OK
Hydrocortison (HCS)	H0888	FDA approved	DMF	82.9	100	--	OK	OK	OK
3-Indolepropionic acid (IPA)	220027	Natural	DMF	196	100	--	lethal	OK	OK
Melatonin (MLT)	M5250	Dietary	DMF	198	100	--	OK	OK	OK
Naringin (NRG)	91842	GRAS	DMF	34.5	10	--	--	OK	OK
Pyrogallol (PYG)	P0381	GRAS	DMF	238	100	--	OK	OK	OK
Resveratrol (RSV)	R5010	Natural	DMF	285	100	--	OK	OK	OK
Rosmarinic acid (RMA)	R4033	Natural	DMF	97	100	--	OK	OK	OK
Sesamol (SML)	S3003	Natural	DMF	217	100	--	OK	OK	OK
Silymarin (SLY)	S0292	Natural	DMF	41.5	10	--	--	OK	OK
Sinapic acid (SNA)	D7927	Natural	DMF	44.6	10	--	--	OK	OK
Syringic acid (SA)	S6881	Natural	DMF	80.8	50	--	OK	OK	OK
Syringaldehyde (SYA)	S7602	Natural	DMF	164.8	100	--	lethal	OK	OK
tert-Butylhydroquinone (TBHQ)	112941	Food additive	DMF	198.8 *	100	--	lethal	lethal	OK
Umbelliferon (UBL)	H24003	Natural	DMF	197.5 *	100	--	OK	OK	OK

Disease Models & Mechanisms • Supplementary information

Table S2. Comparison of characteristics and detection protocol for vdbp-NanoLuc and vdbp-mCherry systems.
When compared to the ELISA assay of vdbp-mCherry, the quantification of vdbp-NanoLuc allows a 20-fold reduction in cost and 6-fold reduction in processing time for a 96-well plate.

	**vdbp-NanoLuc**	**vdbp-mCherry**
Protein size	380 a.a.	446 a.a.
Predicted MW	43 kDa	50 kDa
Detection method	Luminometry	ELISA
Cost for a 96-well plate	**$ 35 (Furimazine)**	**$ 700**
Time for a 96-well plate	**20 min**	**120 min**
Number of steps for assay	3	8
Assay protocol	Add 50 µL urine/well	Add 50 µL urine/well
	Add 50 µL assay buffer	Add 50 µL assay buffer
	Plate reading	Incubation for 60 min
	--	3 x wash
	--	Add 100 µl TMB solution
	--	Incubation for 10 min
	--	Add 100 µL Stop solution
	--	Plate reading
Inter-assay CV	< 10%	NA
Intra-assay CV	< 5%	NA
